# *Wolbachia*-mediated virus blocking in mosquito cells is dependent on XRN1-mediated viral RNA degradation and influenced by viral replication rate

**DOI:** 10.1371/journal.ppat.1006879

**Published:** 2018-03-01

**Authors:** Saijo Thomas, Jiyoti Verma, Megan Woolfit, Scott L. O’Neill

**Affiliations:** 1 Institute of Vector-Borne Disease (IVBD), Monash University, Clayton, VIC, AUSTRALIA; 2 Infection and Immunity Program, Biomedicine Discovery Institute and the Department of Biochemistry and Molecular Biology, Monash University, Clayton, VIC, AUSTRALIA; Institut Pasteur, FRANCE

## Abstract

*Wolbachia* is currently being developed as a novel tool to block the transmission of dengue viruses (DENV) by *Aedes aegypti*. A number of mechanisms have been proposed to explain the DENV-blocking phenotype in mosquitoes, including competition for fatty acids like cholesterol, manipulation of host miRNAs and upregulation of innate immune pathways in the mosquito. We examined the various stages in the DENV infection process to better understand the mechanism of *Wolbachia*-mediated virus blocking (WMVB). Our results suggest that infection with *Wolbachia* does not inhibit DENV binding or cell entry, but reduces virus replication. In contrast to a previous report, we also observed a similar reduction in replication of West Nile virus (WNV). This reduced replication is associated with rapid viral RNA degradation in the cytoplasm. We didn’t find a role for host miRNAs in WMVB. Further analysis showed that the 3’ end of the virus subgenomic RNA was protected and accumulated over time suggesting that the degradation is XRN1-mediated. We also found that sub genomic flavivirus RNA accumulation inactivated XRN1 in mosquito cells in the absence of *Wolbachia* and led to enhancement of RNA degradation in its presence. Depletion of XRN1 decreased WMVB which was associated with a significant increase in DENV RNA. We also observed that WMVB is influenced by virus MOI and rate of virus replication. A comparatively elevated blocking was observed for slowly replicating DENV, compared to WNV. Similar results were obtained while analysing different DENV serotypes.

## Author summary

Dengue virus (DENV) is a human pathogen transmitted by *Aedes* mosquitoes. Infection with DENV causes dengue fever and may develop into life-threatening dengue hemorrhagic fever. Dengue disease is increasing globally and current control methods are proving ineffective in curtailing this growing problem. A novel strategy to stop DENV transmission is currently being trialled in five countries through the introduction of *Wolbachia*, an insect endosymbiont, into wild *Aedes aegypti* populations. Various mechanisms have been proposed to explain *Wolbachia*-mediated virus blocking (WMVB) including the response of the host to *Wolbachia* and factors like cholesterol, immune genes and miRNAs. Here we followed the fate of virus in mosquito cell lines and found that *Wolbachia* does not alter virus binding or internalisation. Further tracking of the virus shows that its replication is reduced in the presence of *Wolbachia*. The reduced replication is associated with increased viral RNA degradation for both DENV and West Nile virus (WNV). Unlike earlier reports, we didn’t find any evidence for miRNA involvement in WMVB. Analysing the viral RNA further shows that the 3’ region of viral RNA is not fully degraded, indicating that the degradation is likely due to the cellular enzyme XRN1. Accumulation of DENV 3’ regions inhibited XRN1 in the absence of *Wolbachia* and reduced the activity of XRN1 but not in the presence *Wolbachia*. Knockdown of XRN1 using siRNA resulted in decreased WMVB associated with increased DENV RNA. The magnitude of WMVB is also dependent on the infectious virus dose and the intrinsic rate at which the virus strain replicates. Similar results were seen for different DENV serotypes confirming that slowly replicating viruses are blocked more efficiently by *Wolbachia*.

## Introduction

Dengue is the most important mosquito-transmitted viral disease of humans in terms of the global burden of disease[1]. Current control methods focus almost entirely on vector control through either preventative source reduction or insecticide spraying in response to outbreaks. The increasing global incidence of dengue is a testament to the shortcomings of the current approach to dengue control. The introduction of the endosymbiotic bacterium *Wolbachia pipientis* into *Ae*. *aegypti* has been shown to interfere with the replication of RNA viruses like dengue (DENV), Chikungunya virus (CHIKV), Yellow fever virus (YFV), West Nile virus (WNV), Semliki Forest Virus (SFV) and Zika virus [2–6] and thus potentially reduce their transmission by mosquitoes.

A number of mechanisms have been proposed to contribute to *Wolbachia-*mediated virus blocking (WMVB). The presence of *Wolbachia* has been shown to up regulate reactive oxygen species (ROS)-dependent activation of Toll pathway genes and associated anti-microbial effectors as well as genes involved in melanization and methyltransferase [7–9]. Studies in *D*. *melanogaster* flies and cell lines, however, have shown that WMVB is independent of the Toll, Imd and RNAi pathways, indicating that immune activation is not required for blocking, though it may enhance it [5, 10, 11].

Alternatively, *Wolbachia* may compete with viruses for key host intracellular molecules such as fatty acids, especially cholesterol or amino acids, thus reducing viral replication [12, 13]. Finally, WMVB may be mediated via manipulation of the expression of host miRNAs. The miRNA aae-mir-2940, for example, is highly expressed in both mosquitoes and cell lines infected with *Wolbachia* and reduces the expression of AaDnmt2 and induced the expression of metalloprotease gene that affects the replication of *Wolbachia* and viruses [14, 15]. On the other hand, *Wolbachia* is able to block viral replication in *D*. *melanogaster* Jw18 cells without upregulating host miRNAs [5].

The exact mechanism responsible for WMVB remains unknown. Previous studies have typically examined the response of the host to the presence of *Wolbachia* to attempt to dissect the mechanism of WMVB. Here, we have instead investigated the fate of the virus itself.

We tracked the different stages of virus replication including its binding to cellular receptors, internalisation, replication and egress in the *Ae*. *aegypti*-derived Aag2 cell line [16, 17]. Our study showed that WMVB is not associated with inhibition of virus binding or internalisation. We further analysed the fate of viral RNA and found that it degrades and that the 3’ end of the virus subgenomic RNA accumulates over time, indicating the potential involvement of exoribonuclease XRN1. sfRNA accumulation also inhibited XRN1 activity and in turn enhanced XRN1 mediated degradation in the presence of *Wolbachia*. The presence of *Wolbachia* does, however, efficiently block the replication of WNV and all DENV serotypes. This blocking, however, was dependent on the virus MOI and the rate of viral genome replication.

## Results

### *Wolbachia* does not affect DENV binding or internalisation

DENV enters a host cell by binding to its receptors followed by endocytosis [18, 19]. We speculated that *Wolbachia*-mediated receptor blocking or change in receptor expression could decrease viral binding and internalisation. We tested each of these steps separately.

**Fig 1:**
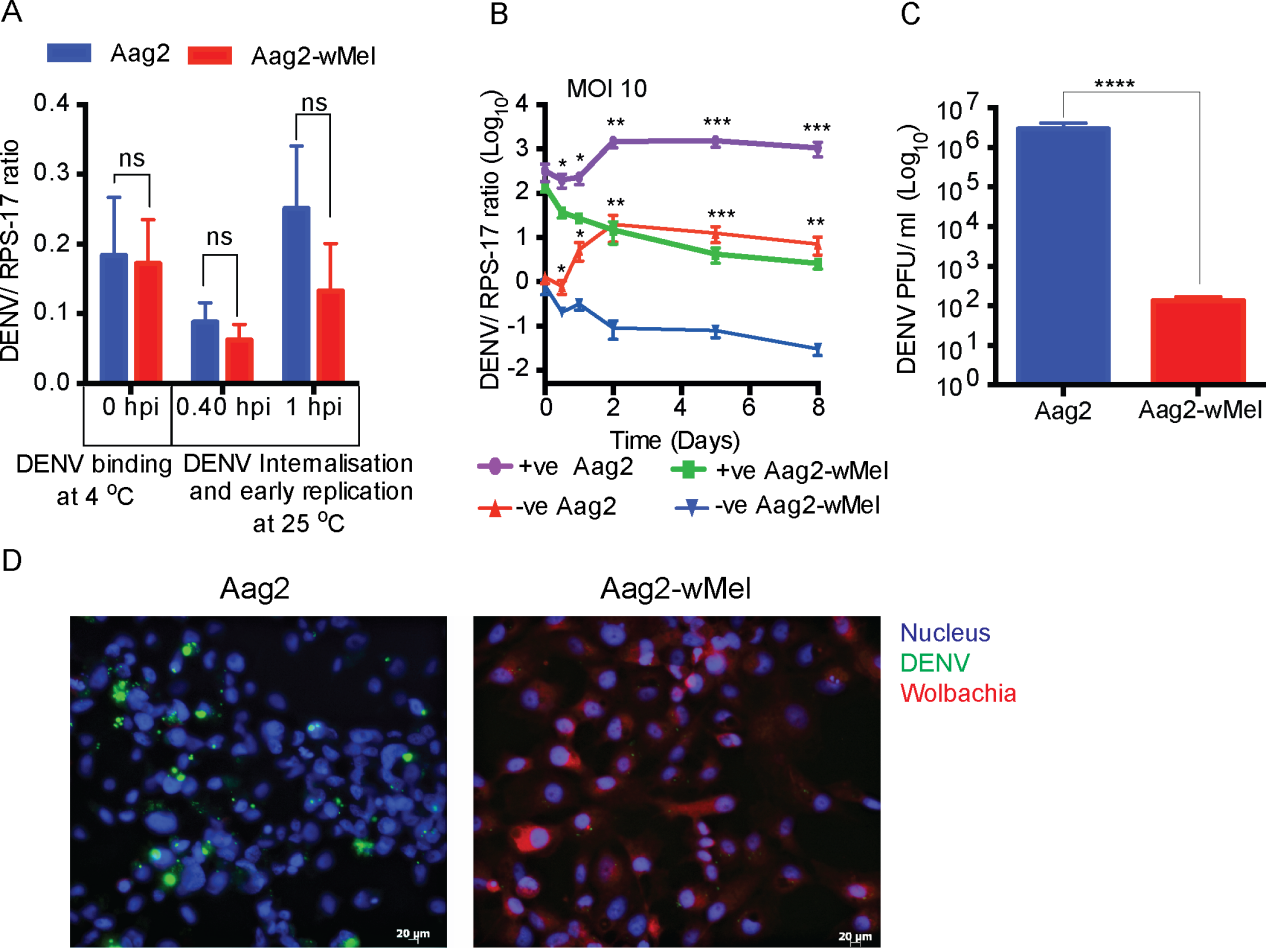
*Wolbachia* does not affect DENV binding or internalisation but viral RNA replication. (A) Aag2 and Aag2-*w*Mel cells were infected with DENV (MOI = 1) and collected at different time points starting from 0 hpi (virus binding) to 1 hpi (virus replication). DENV RNA levels determined from total cellular RNA through quantitative RT-qPCR using primers DENV-G-F, DENV-G-R and DENV-G- FAM Probe and normalised to RPS-17 RNA levels using primers Rps17_TaqM_FW, Rps17_TaqM_RV, and rps17-LC640 probe (n = 3). (B) Strand-specific analysis of *Wolbachia*-mediated RNA degradation in DENV and WNV. Aag2 and Aag2-*w*Mel cells were infected with DENV (MOI = 10) and DENV positive and negative strands were quantified at different time points from total cellular RNA (n = 3). (C) At 5 dpi with DENV, cell culture supernatant was harvested and assayed for viral titre by plaque assay and plotted as plaque forming units/ml (n = 3). All Data are expressed as mean ± SEM (n = 3). * *P* ≤ 0.05 **P ≤ 0.01, *** *P* ≤ 0.001, ****P ≤ 0.0001. (D) Immunofluorescence microscopy of Aag2 and Aag2-*w*Mel cells infected with DENV at an MOI of 5 and analysed 24 hpi. DENV is labelled in green, *Wolbachia* in red and nucleus in blue.

To test whether the presence of *Wolbachia* affects the number of virus particles bound to cellular receptors, we incubated DENV with Aag2 and Aag2 cells containing the *w*Mel strain of *Wolbachia* (Aag2-*w*Mel) at 4°C for 1 hour. At this temperature, the virus can bind to cellular receptors, but is not internalised [20, 21]. After washing away unbound viral inoculum, we quantified DENV RNA (Fig 1A) and found that levels of DENV do not differ significantly on cells with and without *Wolbachia*. This suggests that the presence of *Wolbachia* does not influence the attachment of virus to cells. The experiment was repeated at different MOIs of DENV, and also with WNV, and similar results were obtained (S1 Fig) in all cases.

We then tested whether *Wolbachia* affects DENV internalisation by raising the temperature to 25°C. At this temperature, the virus enters cells through receptor-mediated, clathrin-dependent endocytosis, which allows for virus uncoating and replication [18, 19]. We quantified intracellular DENV RNA at 25 minutes and 1 hour post-infection and found viral levels were similar in Aag2 and Aag2-*w*Mel cells at both time points (Fig 1A), indicating that virus internalisation and early replication was not affected by *Wolbachia*.

### *Wolbachia* strongly inhibits DENV replication

After internalisation by endocytosis and uncoating, the DENV RNA genome is released into the cytoplasm, and the process of viral RNA replication starts at the rough endoplasmic reticulum. We tracked viral replication by quantifying DENV RNA at multiple time points across 8 days’ post-infection. Significant differences in DENV RNA levels between Aag2 and Aag2-*w*Mel cells became apparent almost immediately (Fig 1B). As early as 0.5 days post-infection (dpi), cell lines with *Wolbachia* contained ~2- to ~5-fold less intracellular DENV RNA copies than Aag2 cells. By day 5, the difference between cell types had increased to up to 388-fold (Fig 1B). This pattern of DENV increase in Aag2 cells and decrease in Aag2-*w*Mel cells is also observed for negative strand RNA (Fig 1B). In parallel, DENV titre in the media, after its egress from the cell, reached ~10^6^ PFU/ml in Aag2 cells, but only ~10^2^ PFU/ml in the presence of *Wolbachia* (Fig 1C). Analysis of DENV infected cells using immunofluorescence microscopy also shows that *Wolbachia* reduces DENV replication (Fig 1D). This is evident from the absence of non-structural protein 1 (NS1) in the presence of *Wolbachia* in Aag2-wMel cells and further supports the observation that *Wolbachia* strongly diminishes DENV replication.

Increasing differences in DENV levels were not only due to replication of DENV in Aag2 cells, but also to a steady decrease in DENV in Aag2-*w*Mel cells over time. At 0.5 dpi, DENV levels in Aag2-*w*Mel cells were ~6-fold less than the primary inoculum at 0 dpi. By days 5 and 8 post-infection, DENV levels were ~40 to 60-fold lower than at day 0, respectively (Fig 1B). These results suggest that the viral RNAs which are unable to engage in replication are subjected to degradation in the presence of *Wolbachia*. The experiments were also repeated at an MOI of 1 and similar results were observed (S2A Fig).

To confirm that above results are not cell-line specific, we repeated the experiment using RML-12 cells of *Aedes albopictus* origin, and observed a similar steady decrease of DENV in RML-12-*w*Mel cells over time (S3 Fig).

### DENV RNA undergoes degradation to produce subgenomic flavivirus RNA

**Fig 2:**
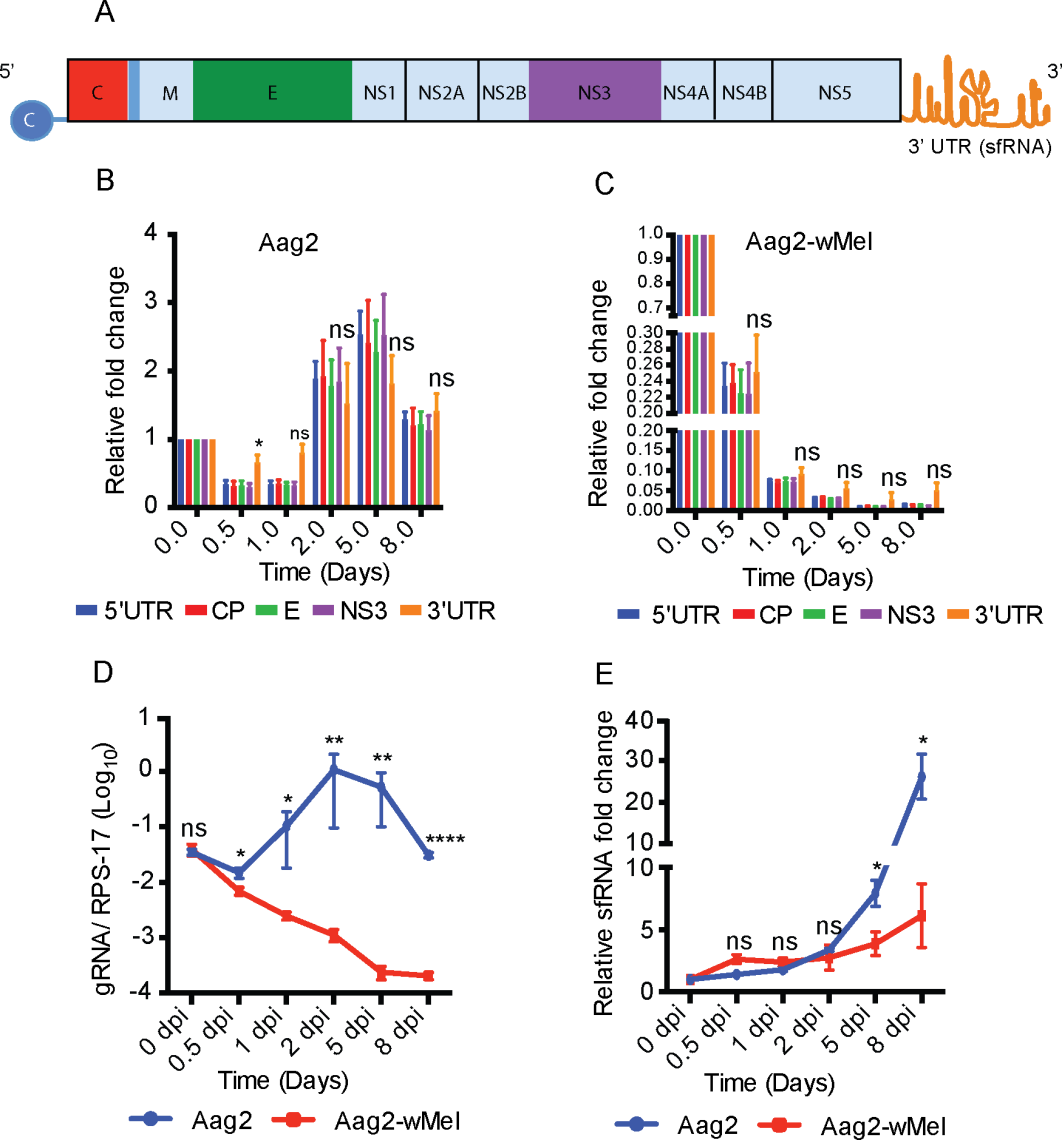
DENV RNA is quickly degraded in the presence of *Wolbachia*. (A) Schematic representation of the DENV genome with its 5’ & 3’ UTRs and different DENV proteins. The colours (other than pale blue) of genomic regions correspond to bars in graphs in B and C. (B and C) Aag2 and Aag2-*w*Mel cells were infected with DENV-2 (MOI = 10) and collected at different time points as shown. Total cellular RNA was used to analyse RNA degradation in different regions of the DENV genome (colour coded) from 5’ UTR to 3’ UTR through quantitative RT-qPCR and normalised to RPS-17 RNA levels. Data are analysed by comparing the 5’UTR with 3’UTR and expressed as mean ± SEM (n = 3). (D) Ratio of gRNA normalised to RPS-17 RNA levels at different time points after DENV infection. (E) Analysis of subgenomic RNA in DENV-infected Aag2 and Aag2-*w*Mel cells. Data are shown as relative fold change at different time points after DENV infection. All Data are expressed as mean ± SEM (n = 3). ns: not significant, * *P* ≤ 0.05 **P ≤ 0.01, *** *P* ≤ 0.001, ****P ≤ 0.0001.

To further analyse the degradation process of viral RNA, we quantified DENV RNA using primers that span different regions of the DENV genome (Fig 2A). The increase in all regions of DENV genomic RNA correlates well with an increase in viral replication after 1 dpi in Aag2 cells (Compare Fig 1B with Fig 2B). In contrast, in the presence of *Wolbachia*, there was a substantial and consistent decrease in the levels of all regions of the DENV genome over time (Fig 2C). There were no significant differences in the rate of accumulation of any region of the DENV genome, with the exception of 0.5 dpi in Aag2 cells, where the levels of 3’ UTR were significantly higher than other regions (Fig 2B). We also observed minor non-significant increase in 3’ UTR at all time points in Aag2-*w*Mel cells (Fig 2C).

The 3’ UTR of flavivirus genomes produces subgenomic flavivirus RNA (sfRNA) as a by-product of incomplete RNA digestion by XRN1[22–27]. This Subgenomic RNA accumulates in cells and is an important factor in flavivirus pathogenicity [28]. Since the primers used above could detect only genomic RNA, we next examined levels of 3’ UTR at different time points using primers that detect both gRNA and sfRNA.

Consistent with our previous results, DENV 3’ UTR gRNA levels increased in Aag2 cells over time, but steadily decreased in Aag2-*w*Mel cells (Fig 2D). In contrast, sfRNA levels increased over time in both cell types, although the increase was far less dramatic in the presence of *Wolbachia* (only ~6-fold compared to ~26-fold higher at 8 dpi; Fig 2E). This happens due to incomplete RNA digestion by XRN1 were sfRNA is not digested. As a result, levels of sfRNA increase over time, but not the gRNA, which is sensitive to XRN1 and gets degraded. It should also be noted that WMVB does not completely inhibit virus replication as is evident from the low levels of positive and negative strands (Fig 1B) throughout the time course. This also explains the reduced accumulation of sfRNA in Aag2-*w*Mel cells.

### WMVB prevents inhibition of XRN1-mediated degradation

Accumulation of sfRNA in DENV-infected cells can lead to inactivation of the exoribonuclease XRN1, thereby increasing the overall stability of viral and host RNAs [29]. The much lower accumulation of sfRNA in Aag2-*w*Mel cells compared to Aag2 cells raises the possibility that reduced levels of sfRNA might not be sufficient to inhibit XRN1 in infected cells. If so, this could contribute to a positive feedback cycle supporting *Wolbachia*’s ability to reduce dengue RNA levels.

**Fig 3:**
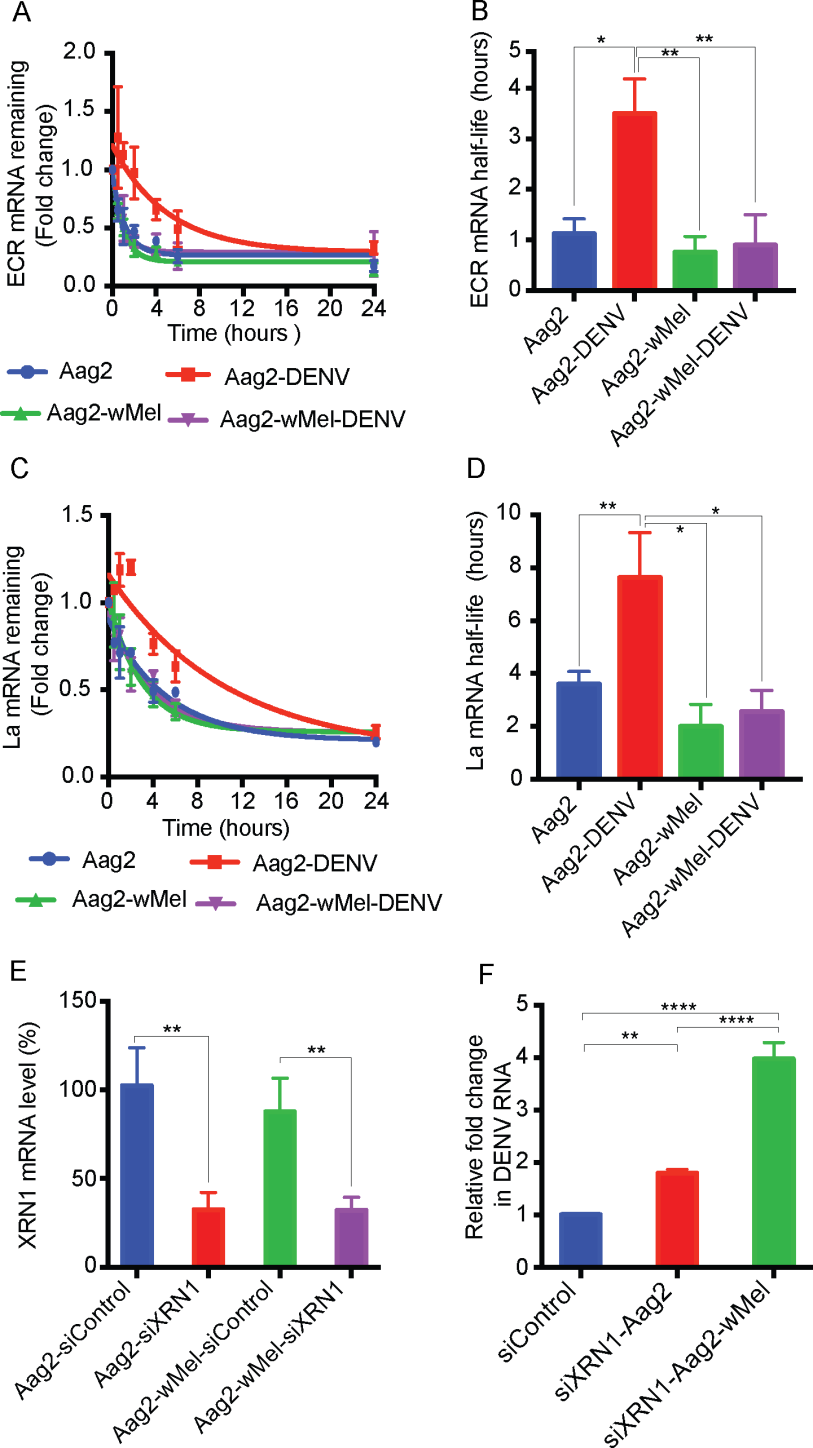
Role of XRN1 in WMVB. (A-D) Insect Cellular mRNA stability is altered by DENV sfRNA. The half-life of ECR (A & B) and La (C&D) mRNA was determined in Aag2 and Aag2-*w*Mel cells mock infected or infected with DENV after Actinomycin-D treatment by RT-qPCR. Data are expressed as mean ± SEM (n = 3), * *P* ≤ 0.05 **P ≤ 0.01. (E & F) Effect of XRN1 depletion on WMVB. Aag2 and Aag2-*w*Mel cells were transfected with XRN1 specific siRNAs (siXRN1) or control (siCtrl) twice followed by infection with DENV at an MOI of 0.1. The cells were lysed after 48 h and viral RNA was quantified by RT-qPCR. (E) RT-qPCR showing the depletion of XRN1 RNA, 48 h after the siRNA transfection. (F) Relative intracellular DENV RNA abundances 48 h after infection. Data are expressed as mean ± SEM (n = 3). * *P* ≤ 0.05, **P ≤ 0.01, ****P ≤ 0.0001 and compared using one-way ANOVA.

To determine the effect of *Wolbachia* on RNA decay, we tested degradation of host mRNA during dengue infection [29, 30]. Aag2 and Aag-*w*Mel cells, mock or infected with DENV, were treated with actinomycin D to stop cellular transcription. We then estimated the half-life of the transcripts of two host genes, ECR and La, which have previously been studied in the insect cells for mRNA decay [31]. In mock-infected Aag2 cells, the half-life of La and ECR (3.60 ± 0.28 and 1.13 ± 0.17 hours, respectively; Fig 3A–3D) were similar as previously reported [31]. DENV infected Aag2 cells showed approximately twofold increase in the half-life of both La and ECR transcripts reaching 7.63 ± 0.98 and 3.49 ± 0.40 hours, respectively (Fig 3A–3D) consistent with an increased accumulation of DENV-derived sfRNA inhibiting XRN1. However, in DENV infected Aag2-*w*Mel cells, this increase in half-life of host RNAs was not observed, suggesting that DENV is unable to inhibit Xrn1 activity in the presence of *Wolbachia*. It should be also noted that inhibition of XRN1 stabilises various cellular RNAs and results in changes to cellular gene expression. This may be also a strategy by flaviviruses to escape cellular surveillance mechanisms that can otherwise inhibit their replication [29, 30].

XRN1 is known to be associated with DENV replication regulation and its downregulation can positively enhance virus replication [32]. To investigate this further, we directly tested the effect of DENV RNA following siRNA knockdown of XRN1. We observed ~70% reduction in XRN1 mRNA levels when XRN1 specific siRNAs were used (Fig 3E). In Aag2 cells, XRN1 knockdown resulted in ~ 2-fold increase in DENV RNA (Fig 3F) as expected. Whereas XRN1 knockdown showed a greater increase in DENV RNA levels (~4-fold, p < 0.0001) in Aag2-*w*Mel cells (Fig 3F). These results further support our observation that XRN1 activity is not fully blocked in Aag2-*w*Mel cells. Thus, an interaction between *Wolbachia*, viral RNA and XRN1 may be central to WMVB.

### *Ae*. *aegypti*-derived miRNAs levels are not altered by *Wolbachia*

**Fig 4:**
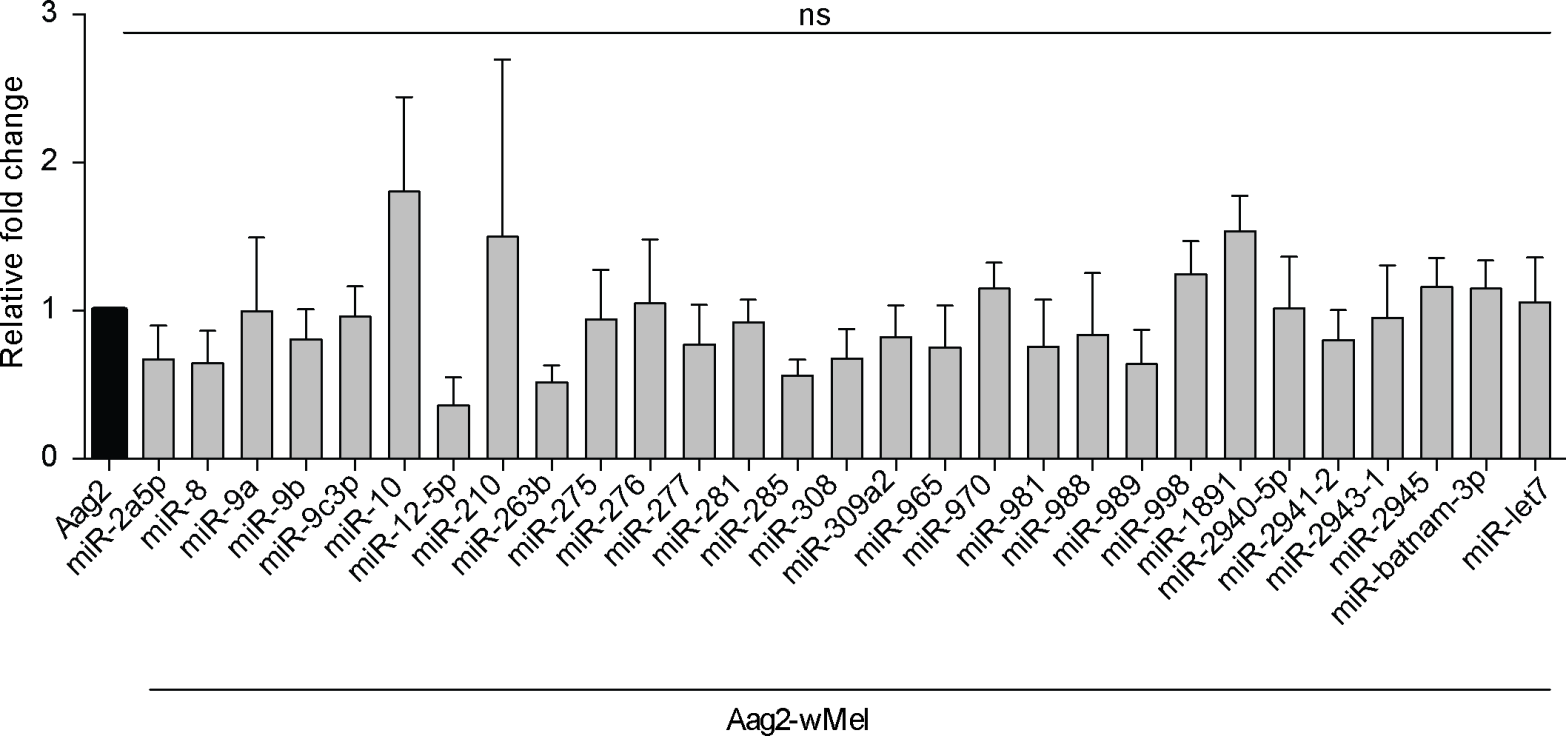
Expression of miRNAs due to *Wolbachia* in Aag2 cells. The expression levels of host miRNAs were analysed in Aag2 and Aag2-*w*Mel cells through RT-qPCR and normalised with RPS-17. Data are expressed as mean ± SEM (n = 3).

Even though lower levels of sfRNA accumulated in DENV-infected Aag2-*w*Mel cells, we observed a considerable amount of degradation of the viral 3’ UTR region (Fig 2C). This could be caused by other RNA-degrading factors, including miRNAs even if RNA is eventually degraded by XRN1 and exosome [33]. Previous studies have suggested that upregulation of some host miRNAs is an important factor in WMVB [14, 15]. We used qPCR to analyse the expression of a number of host miRNAs that have either been previously reported to be upregulated in the presence of *Wolbachia* [14, 15] or which we identified as having the potential to bind to the 5’ or 3’ UTR of the DENV genome (S2 Table). We saw no significant change in the expression of any of these miRNAs in the presence of *Wolbachia* (Fig 4), suggesting that they are not involved in *Wolbachia*-mediated inhibition or degradation of viral RNA. Our results support similar observations recently reported for Drosophila cells, in which host miRNA expression was not affected by *Wolbachia* [5].

### *Wolbachia* also reduces replication of West Nile virus

**Fig 5:**
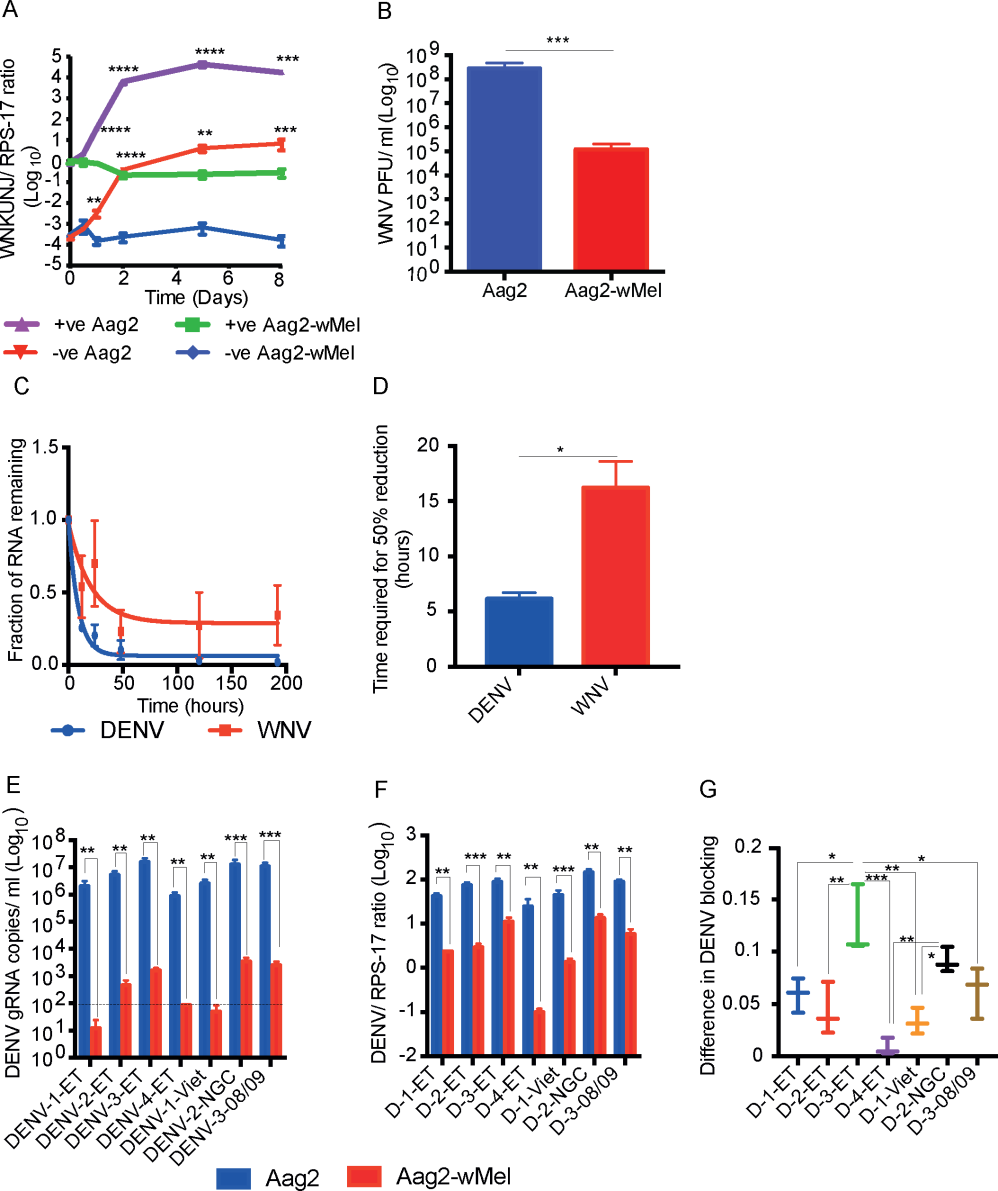
Intrinsic virus replication rate is a determinant in WMVB. (A & B) WNV replication is inhibited by *Wolbachia*. (A) WNV positive and negative strands were quantified through RT-qPCR at different time points after infecting cells (MOI = 10), (n = 5). (B) At 5 dpi with WNV, cell supernatant was harvested and assayed for viral titre by plaque assay (n = 5). (C & D) Aag2-wMel cells infected with DENV and WNV were compared through one phase decay up to 8 dpi. (E-G) DENV blocking by *Wolbachia* is not serotype dependent. Aag2 and Aag2-*w*Mel cells were infected with various DENV isolates from serotype 1–4 at an MOI of 1 and analysed 5 dpi. (E) Viral RNA was isolated from cell culture supernatant and the number of virus copies were calculated using RT-qPCR. Dotted line denotes the minimum detection limit. (F) Total cellular RNA from cells were used to detect DENV using RT-qPCR and normalised with RPS-17. All Data are expressed as mean ± SEM. **P ≤ 0.01, *** *P*≤ 0.001. (G) Difference in blocking of different serotypes calculated from the ratio of DENV in Aag2-*w*Mel cells divided by the ratio of DENV in Aag2 cells (Aag2-*w*Mel/Aag2). Data are expressed as mean ± SEM (n = 3). * *P* ≤ 0.05, **P ≤ 0.01, ****P ≤ 0.0001 and compared using one-way ANOVA.

As it has previously been reported that intracellular West Nile virus replication is enhanced by *Wolbachia* [4], we also investigated the replication of this virus in our system. As expected, after infecting *Wolbachia*-free cells with an MOI of 10, the titre of WNV of both polarities increased over time, with RNA levels peaking at 5 dpi (Fig 5A).

In contrast to the previous report [4], however, we did not see an increase in WNV RNA levels in the presence of *Wolbachia*. Instead, we observed that intracellular WNV RNA copies were reduced for both positive and negative strands (~7-fold and ~6-fold reduction at 8 dpi compared to 0 dpi). The difference in positive strand WNV RNA levels between cells with and without *Wolbachia* was ~46-fold at 1 dpi and ~4×10^5^-fold at 8 dpi. Analysis of cell culture supernatant showed similar results, with WNV titre reaching ~10^8^ PFU/ml at 5 dpi in Aag2 cells, but only ~10^5^ PFU/ml in Aag2-*w*Mel cells.

### WMVB is dependent on virus MOI and intrinsic replication rate

We observed differences between DENV and WNV in the extent of its replication (compare Figs 1B, 1C, 5A and 5B). Early inhibition at 0.5 dpi in the presence of *Wolbachia*, as seen for DENV, was not seen for WNV at the same MOI. Similarly, we saw only ~7-fold overall reduction from the initial inoculum of WNV in Aag2-*w*Mel cells at 8 dpi, compared to a ~60-fold reduction of DENV. Similar results were observed at different MOIs (S2C–S2F Fig). WNV reached a titre of ~10^8^ PFU/ml in Aag2 cell culture supernatant after its egress, compared to ~10^6^ PFU/ml with DENV on 5dpi. This difference was further analysed by comparing the WMVB of DENV and WNV through one phase decay up to 8 dpi. Even though both viral RNAs were degraded during WMVB, the time to reach 50% viral RNA reduction differed (Fig 5C and 5D). A 50% reduction of DENV RNA was seen as early as ~ 6 hours compared to ~ 16 hours for WNV. This can be attributed to the difference in the rate of replication of both viruses. In DENV, due to its slower replication, new viral RNAs are not synthesised at the same rate as in WNV. This results in fast depletion of viral RNA resulting in 50% degradation happening as early as ~ 6 hours. RNA degradation occurs similarly in WNV but due to its comparatively fast replication, new RNA strands are synthesised at the same time. This also further extends the 50% reduction time for WNV RNA from primary inoculum.

### *Wolbachia* blocks DENV irrespective of its serotype

We next wanted to confirm variation in WMVB and its relationship with intrinsic replication rate by comparing different strains of DENV virus from different serotypes, which might differ in the rate of replication but still utilise a similar replication mechanism. We first analysed the intrinsic replication rate of DENV serotypes 1–4, all passaged up to 7 times in C6/36 cells and infected Aag2 and Aag2-*w*Mel cells at an MOI of 1. Analysis of virus copy number at different time points showed that virus replication was different for all these four serotypes (S4 Fig). Regardless of serotype differences in DENV, they all were inhibited in the presence of *Wolbachia*. DENV-3 ET which reached the highest titre of ~10^7^ genome copies/ ml showed the lowest WMVB (S4 Fig).

To analyse this further, we then compared DENV-1 Viet, DENV-2 NGC and DENV-3 08/09 along with DENV 1–4 ET isolates. All the virus infection, RNA isolation and quantification were carried out at the same time. Analysis of virus copies from cell culture supernatant and intracellular RNA after infecting Aag2 and Aag2-*w*Mel cells shows the extent of intrinsic virus replication (Fig 5E and 5F). Viral RNA in cell culture supernatant reached a titre of ~10^7^ genome copies/ ml for DENV-3 ET, DENV-2 NGC and DENV-3 08/09 compared to DENV-1 ET, DENV-2 ET, and DENV-4 ET and DENV-1 Viet which reached a slightly low titre of ~10^6^ genome copies/ ml. Again, regardless of serotype difference, all seven DENV isolates were inhibited in the presence of *Wolbachia* with a higher viral inhibition observed among DENV-1 ET, DENV-4 ET and DENV-Viet which were below the detection limit of 10^2^ genome copies/ ml in cell culture supernatant. Intracellular analysis of viral RNA also showed similar results (Fig 5F). The greatest difference in blocking was observed between DENV-3 ET and DENV-4 ET (*** p < 0.0001, Fig 5G) which, grew to the highest and lowest copies respectively. These results show that *Wolbachia* blocks DENV irrespective of its serotype and that the WMVB is influenced by the intrinsic replication rate.

## Discussion

The insect endosymbiont *Wolbachia* is currently being used as a tool to reduce transmission of dengue and other *Aedes* transmitted viruses [34]. Even though various studies have been carried out to understand the exact mechanism behind WMVB [7, 8, 12, 14], none has examined the detailed virus infection process in the *Wolbachia* context. DENV, a positive-stranded RNA virus enters the host cell through host receptors and releases its RNA into the cytoplasm where it is translated by host ribosomes. The translated products include polyproteins encoding the replication machinery used to produce positive and negative RNA strands [35, 36]. In the current study, we saw no effect of *Wolbachia* on the early stages of virus binding or cellular internalisation. Instead, we found that *Wolbachia* reduced virus replication that is followed by enhanced viral RNA degradation.

RNA viruses use different strategies to stabilise transcripts and evade the cellular RNA decay machinery and so maintain a continuous infection. In flaviviruses, some of the RNAs produced are degraded through a 5’ to 3’ exonucleolytic pathway [37, 38]. This process leads to an accumulation of sfRNA due to incomplete degradation. This XRN1-mediated degradation typically stalls near the 3’UTR, and leads to inhibition of the XRN1 enzyme, as it remains bound to sfRNA. This inhibition has been reported before in mammalian cells [29, 30] and now in insect cells to cause increased stabilisation of cellular mRNA and thus giving a better environment for the survival of viral RNAs. In the presence of *Wolbachia*, we observed that DENV replication is inhibited and thus doesn’t lead to accumulation of sfRNA making the cells not ideal for virus growth, as an active XRN1 enzyme will be regulating the RNA level.

We are not assuming that XRN1 mediated viral RNA degradation is the only mechanistic explanation for WMVB but our results suggest that it plays an important part. The role of other decay factors associated with XRN1 including UPF1, SMG5, SMG6 and SMG7 also cannot be ruled out [39]. It is now well established along with this work that *Wolbachia* inhibits replication of most of the positive stranded RNA viruses. This is not the case with negative stranded RNA viruses such as Phasi Charoen-like bunyavirus which are unaffected by the presence of *Wolbachia* [40]. It is also known that depletion of RNA decay factors like XRN1 and UPF1 doesn’t have any effect on infection of negative stranded RNA viruses of the *Paramyxoviridae* and *Bunyaviridae* families [41–43]. Furthermore, they cap-snatch the 5′ end of host mRNAs using a virally encoded endonuclease. This makes the 5′ end of the viral mRNA indistinguishable from endogenous mRNAs and helps in protection from degradation [43, 44]. These differences between positive and negative stranded viruses and the correlation with WMVB further suggests the significance of viral RNA decay in contributing to WMVB.

Comparison of the extent of WMVB by comparing WNV with DENV or by comparing different strains of DENV that show variation in replication rate and subsequently the titre they attain at a given time point, shows that the degree of WMVB is highest with slower replicating viruses. Ideally, RNA viruses should replicate quickly and be recruited to cellular membranes to evade the host RNA decay machinery. A slowly replicating virus is more prone to RNA degradation [41, 45, 46], which can occur rapidly: the half-life of a cellular mRNA is on average only hours [47]. Hence, slowed replication in the presence of *Wolbachia* should make viral RNA more prone to host RNA decay mechanisms, resulting in viral RNA degradation. At higher viral MOI, the presence of *Wolbachia* may prevent an equally high rate of replication, leading to sudden viral RNA degradation as we observed for both DENV and WNV. Thus, it is possible that *Wolbachia* may block slowly replicating virus more efficiently than viruses that replicate rapidly to higher titre. We predict, therefore, that a slowly replicating virus should be more severely affected by WMVB. As an example, DENV, which grows relatively slowly and to lower titre (~10^6^ PFU/ml) is more efficiently blocked by *Wolbachia* compared to WNV, which grows robustly and to a titre of (~10^8^ PFU/ml).

In addition to the extent of virus replication, WMVB is directly affected by *Wolbachia* density in the host [48, 49]. It is thus possible that competition for shared cellular resources may be exacerbated at higher densities, increasing the negative impact of *Wolbachia* on viral replication and subsequent RNA degradation by XRN1.

## Materials and methods

### Cell lines, virus propagation and titration

Aag2-*w*Mel cells containing the *w*Mel strain of *Wolbachia* were prepared by infecting Aag2 cells with *wMel* (S1 Text). Aag2-Tet (hereafter referred as Aag2) was prepared by treating Aag2-*w*Mel cells with tetracycline [49]. Cells were maintained in complete medium containing 44% Schneiders media (Life Technologies), 44% M&M (CaCl_2_ 0.151 gm_,_ MgCl_2_ 0.047, KCl 0.2 gm, NaCl 7 gm, NaH_2_PO_4_ 0.174 gm, Glucose 4 gm, Yeast extract 5 gm, Lactalbumin hydrolysate 6.5 gm, and NaHCO_3_ 0.12 gm per litre) 10% fetal bovine serum (FBS) (Gibco) and 1% penicillin-streptomycin solution (Gibco).

C6/36 cells of *Aedes albopictus* origin were supplied by the European Collection of Cell Cultures (ECACC; Salisbury, United Kingdom) and was purchased from CellBank Australia (Westmead, NSW, Australia) and were grown in RPMI media (Gibco) containing 10% FBS, 1X glutamax (Gibco), 20 mM HEPES (pH 6.9) and 1% penicillin-streptomycin solution (Gibco). Vero cells of African green monkey kidney origin were supplied by the ECACC and was purchased from CellBank Australia and were grown in DMEM medium (Gibco) containing 5% FBS and 1% penicillin-streptomycin solution (Gibco). Cell lines were routinely screened for any *Mycoplasma* contamination. *Wolbachia* densities in cell lines were measured by qPCR and FISH (S1 Text, S6 Fig). Cell lines were also screened for any other flavivirus infection as described before (S1 Text, S7 Fig)

Dengue virus (DENV) 2 ET 300 was passaged up to 7 times in C6/36 cells, and West Nile virus (WNV) (Kunjin subtype) of unknown passage history was further propagated in C6/36 cells [50]. The viruses used for infection were produced after infecting C6/36 cells containing RPMI media (Gibco) with 2% FBS, 1X glutamax (Gibco), and 20 mM HEPES (pH 6.9), and harvested 5 dpi. The harvested virus was aliquoted and stored at -80°C until use.

Virus titre in cell culture media was measured by plaque assay as reported previously [51]. Briefly, Vero cells were seeded towards 90% confluency and infected with the virus of different dilutions. 1% carboxymethyl cellulose containing DMEM medium, 2% FBS and 1% penicillin-streptomycin was added as an overlay and incubated at 37°C for 5–7 days. Plaques were visualised by crystal violet staining.

### Virus binding and infection

Aag2 and Aag2-*w*Mel cells seeded in 12 well plates were incubated at 4°C for 5 minutes, followed by washing once with cold PBS. Cells were infected with DENV-2 while kept on ice at specified MOI and further incubated for 1 hour at 4°C with gentle rocking every 15 minutes. Virus inoculum was then removed and the cells washed three times with cold PBS. Cells were then collected and stored in -80°C till use.

For the internalisation study, media at 25°C was added to the cells followed by continued incubation at 25°C. Cells were washed at each time point with acid glycine (pH 3) followed by PBS (pH 7.4) wash. Cells were collected at different time points and stored in -80°C until use.

All other virus infection experiments were carried out by infecting cells at specified MOI and incubating for 1 hour at 25°C followed by washing with PBS (pH 7.4) three times and continued incubation at 25°C.

### RNA extraction, cDNA and qPCR

Unless otherwise stated, all RNA extraction was carried out from cells using Trizol (Life Technologies), according to manufacturer’s instructions. To synthesise cDNA, RNA was DNase (Roche) treated and converted to cDNA using SuperScript III Reverse Transcriptase (Invitrogen) according to manufacturer’s instructions. RNA integrity was analysed through bioanalyser (Micromon, Monash University, Clayton) (S5 Fig). qPCR was run using the LightCycler 480 Probes Master (Roche) in a LightCycler 480 real-time PCR machine.

### Strand-specific analysis of viral RNA

Strand-specific analysis of DENV was done as described previously [52] using LightCycler 480 SYBR Green I Master (Roche) with 45 cycles followed by melt curve analysis and was normalised with RPS-17.

To analyse West Nile virus RNA strands, positive strand cDNA was synthesised using primer TAGWNKUNJ-E-R and for the negative strand with TAGWNKUNJ-E-F. qPCR was performed using LightCycler 480 SYBR Green I Master (Roche) and with primers Tag_only and WNKUNJ-E-F for the positive strand. To detect negative strands, primers Tag_only and WNKUNJ-E-R were used. The PCR conditions were as follows: Step 1, 95°C for 5 min; Step 2, 95°C for 10 s, 60°C for 10 s, 72°C for 10 s with 45 cycles; followed by melt curve analysis. Both strands were normalised with RPS-17.

### Immunofluorescence microscopy

Aag2 cells grown on coverslips in 6 well plates were infected with DENV at an MOI of 5. 24 hpi, cells were fixed with 4% formaldehyde in PBS for 10 min and then permeabilised by incubating with 4% formaldehyde and 0.2% triton-X-100. Cells were then blocked with 1% BSA in PBS-T (0.2% Triton X-100) for one hour at room temperature and probed with anti-NS1-4G4 mouse monoclonal antibody and anti-wsp rabbit polyclonal antibody at a dilution of 1:100 for an hour followed by washing with PBS-T. Secondary antibody anti-rabbit-alexa 488 and anti-mouse alexa 594 were then added and incubated for an hour. Coverslips were further washed with PBS-T and stained with DAPI for 10 minutes, followed by a 10-minute wash in PBST and rinsed briefly in distilled water. After removing excess water, slides were fixed with antifading reagent (ProLong Gold Antifade Mountant, Thermo Fisher) and were viewed using an immunofluorescence microscope (Zeiss Imager.A1).

### RNA degradation analysis

Primers that span from 5’ to 3’ of the DENV genome (Fig 2A, S1 Table) were used to perform qPCR. 1 μg of DNase-treated total RNA was reverse-transcribed using SuperScript III Reverse Transcriptase (Invitrogen) according to the manufacturer’s instructions. qPCR reaction was performed on LightCycler 480 (Roche) using the FastStart Universal SYBR Green Master Rox (Roche) reagent mix. The data analysis was performed using LinReg software [53].

### Analysis of various DENV serotypes for WMVB

DENV-1 ET, DENV-2 ET 300, DENV-3 ET, DENV-4 ET, DENV-1 Viet, DENV- 3 08/09 were all passaged up to 7 times. DENV-2 NGC was produced by *in vitro* transcription from an infectious clone [54] (S1 Text). All viruses were amplified at the same time in C6/36 cells and harvested 5 dpi and stored in -80°C as aliquots until use. Aag2 and Aag2-*w*Mel cells were infected with various DENV isolates at an MOI of 1 and harvested 5 dpi. The virus was isolated from the media using QIAamp Viral RNA Mini Kit (qiagen) and quantified using RT-qPCR using primers DENV-G-F, DENV-G-R and DENV-G- FAM Probe [55]. Virus copy number was calculated from the standard curve generated from serial dilutions of reverse transcribed DENV RNA prepared with carrier RNA. The minimum detection limit was determined as 10^2^ copies [56]. Total cellular RNA was isolated using directzol RNA miniprep kit (zymo research) and quantified by RT-qPCR using primers DENV-G-F, DENV-G-R, DENV-G- FAM Probe and normalised with RPS-17.

### miRNA-binding analysis and quantification

We screened 124 previously annotated *Ae*. *aegypti* miRNAs [57] to identify those with the potential to bind to the 5’ or 3’ UTR of the DENV genome. For each miRNA-UTR pair, we first calculated the minimum free energy (MFE) of the best potential binding site between the sequences, using the program RNA-hybrid [58]. We then used the shuffleseq function within the Emboss package to generate 100 simulated sequences of the same length and base composition as the real miRNA and calculated the MFE of each simulated sequence to the UTR. We considered *Ae*. *aegypti* miRNAs as candidates for binding to the DENV 5’ or 3’ UTR if their MFE for binding was better than 95% of the simulated sequences for that miRNA-UTR pair.

Aag2 and Aag2-*w*Mel cells resuspended in Trizol (Life technologies) were used to purify RNA using Direct-zol RNA MiniPrep kit (Zymoresearch) according to the manufacturer’s instructions, which included on column DNase digestion. RNA was reverse transcribed using miScript II RT Kit (Qiagen) according to manufacturer’s instruction and amplified using LightCycler 480 SYBR Green I Master (Roche) with each reaction involving forward primer sequence of mature miRNA (S1 Table) and universal reverse primer (Qiagen). Additional adenosine sequence was added to the mature miRNA sequence in case it was required to increase the primer Tm value. The PCR condition was as follows: Step 1, 95°C for 10 min; Step 2, 95°C for 10 s, 60°C for 15 s, 72°C for 10 s for 45 cycles with 45 cycles followed by melt curve analysis. The data analysis was performed using LinReg software [53]. miRNAs were further selected according to primer efficiency and expression value and were normalised with RPS-17.

### Subgenomic RNA analysis

Subgenomic flavivirus RNA (sfRNA) of DENV was quantified as described before [22, 26] with modifications. Briefly, primers QG-F and QGSF-R were used to detect genomic RNA, which gives a PCR product of 309 bp. Primers QGSF-F and QGSF-R were used to detect both genomic and sfRNA with a product size of 184 bp (See S7 Fig in [26]. qPCR was performed using LightCycler 480 SYBR Green I Master (Roche) in a LightCycler 480 real-time PCR machine with 45 cycles and melt curve analysis. Relative levels of genomic RNA were calculated by normalising with RPS-17. Relative levels of sfRNA were calculated by normalising product produced from QGSF primers (gRNA + sfRNA) with product produced from QG and QGSF primers (gRNA) and normalised to RPS-17. The expression levels were shown as fold change in sfRNA level at each time point compared to its level at 0 dpi.

### Analysis of mRNA half-life

The Half-life of cellular mRNA was analysed as reported before [29–31]. Briefly, Aag2 and Aag2-*w*Mel cells were mock infected or infected with DENV at an MOI of 0.1. After 5dpi, the media was changed and replaced with media containing actinomycin D (5 μg/ml) and RNA collected after 30 minutes to ensure cellular transcription shutoff. Total cellular RNA was isolated using directzol RNA miniprep kit (Zymo research) and treated with DNase (Roche).

Total RNA isolated was reverse transcribed using random primer (Thermofisher) and abundance of mRNA was analysed through qPCR using the FastStart Universal SYBR Green Master Rox (Roche) reagent mix with primers ECR-F, ECR-R [31], aae La-F, aae La-R and normalised with RPS-17.

### siRNA transfection

siRNAs (Sigma) containing pool against XRN1 (siXRN1) and control siRNA having scrambled sequence (siControl) were transfected in Aag2 and Aag2-*w*Mel cells using Lipofectamine RNAiMAX (Invitrogen) as reported before [59]. Transfections were carried out twice at 24 hour intervals followed by DENV infection at MOI 0.1. The cells were lysed 48 hpi using trizol (Life Technologies) and RNA isolated using Direct-zol RNA isolation kit (zymo research). RNA was DNase (Roche) treated and quantified by RT-qPCR.

### Statistical analysis

Unless noted otherwise, all data are presented as mean ± SEM from at least three biological replicates. All statistical analysis was performed using t-tests with Welch’s correction unless noted otherwise. Graphs were log10 transformed [60, 61] before statistical analysis unless noted otherwise. All analysis and graphs were generated using prism-6 software and considered significant if *P* ≤ 0.05 (*), **P ≤ 0.01, *** *P* ≤ 0.001, ****P ≤ 0.0001.

## Supporting information

S1 Fig Analysis of virus binding at different MOI. (A) Aag2 and Aag2-*w*Mel cells were infected with DENV at an MOI of 10 at 4°C. RNA levels determined from total cellular RNA through quantitative RT-qPCR using primers DENV-G-F, DENV-G-R and DENV-G- FAM Probe and normalised to RPS-17 RNA levels using primers Rps17_TaqM_FW, Rps17_TaqM_RV, and rps17-LC640 probe. Data are expressed as mean ± SEM (n = 3). ns: not significant. (B &C) Aag2 & Aag2-*w*Mel cells were infected with WNV at an MOI of 1 and 10 at 4°C. RNA levels determined from total cellular RNA through quantitative SYBR Green RT-qPCR using primers WNKUNJ3UTR-F and WNKUNJ3UTR-R and normalised to RPS-17 RNA levels using primers Rps17_TaqM_FW, Rps17_TaqM_RV. Data are expressed as mean ± SEM (n = 3). ns: not significant.
(TIF)

S2 Fig Strand-specific detection of *Wolbachia*-mediated RNA degradation in DENV and WNV. (A) Aag2 & Aag2-*w*Mel cells were infected with DENV (MOI = 1) to determine DENV positive and negative strands, levels at different time points from total cellular RNA through quantitative RT-qPCR and normalised to RPS-17 RNA levels. Data are expressed as mean ± SEM (n = 1). (B) At 5 dpi with DENV, cell culture supernatant was harvested and assayed for viral titre by plaque assay and plotted as plaque forming units/ml. (C & E) WNV positive and negative strands, levels quantified through quantitative RT-qPCR after infecting cells MOI = 1, n = 5 (C) and MOI 100, n = 3 (E) and collected at different time points. (D & F) At 5 dpi with WNV, cell supernatant was harvested and assayed for viral titre by plaque assay and plotted as plaque forming units/ml MOI = 1, n = 5 (D) and MOI 100, n = 3 (F). All Data are expressed as mean ± SEM. ns: not significant, * *P* ≤ 0.05 **P ≤ 0.01, *** *P* ≤ 0.001, ****P ≤ 0.0001.
(TIF)

S3 Fig Analysis of DENV RNA degradation in RML-12 cells. RML-12 and RML-12-*w*Mel cells were infected with DENV (MOI = 1) to determine DENV positive and negative strands, levels at different time points from total cellular RNA through quantitative RT-qPCR and normalised to Actin RNA levels (n = 1).
(TIF)

S4 Fig Analysis of WMVB by different DENV serotypes 1–4. Viral RNA was isolated from cell culture supernatant at different time points and the number of virus copies were calculated using RT-qPCR. Dotted line denotes the minimum detection limit. Data are expressed as mean ± SEM (n = 3).
(TIF)

S5 Fig Electrophoretic analysis of total RNA extracts. Purified total RNA samples from Aag2 and Aag2 *w*Mel cells were examined using BioAnalyzer 2100 (Aglient Technologies) to check RNA integrity. Major ribosomal RNA bands are indicated.
(TIF)

S6 Fig Analysis of *Wolbachia* infection in Aag2 cells. (A)*Wolbachia* density was calculated through qPCR as a ratio of the WD_513 gene in *Wolbachia* normalised to host RPS-17 gene. (B) *Wolbachia* was detected through Fluorescence in situ hybridization (FISH) shown in red and nucleus stained with DAPI (Blue).
(TIF)

S7 Fig Screening of Aag2 and Aag2-*w*Mel cells for flavivirus infection. Lane L: 1000 bp DNA ladder; 1: Cell culture supernatant from Aag2; 2: Cell culture supernatant from Aag2-*w*Mel; 3: Water control; 4: Cell culture supernatant from Aag2 cells infected with WNV and harvested 2dpi; 5: Aag2 cells genomic DNA; 6: Aag2-*w*Mel cells genomic DNA. Expected flavirus PCR product is indicated by an arrow.
(TIF)

S8 Fig Expression of XRN1 RNA levels in Aag2 & Aag2-wMel cells infected with DENV. Total cellular RNA five days post DENV infection (MOI = 10) was analysed for expression of XRN1 RNA through quantitative SYBR Green RT-PCR using primers aae-XRN1-Fand aae-XRN1-R normalised to RPS-17 RNA levels. Data are expressed as mean ± SEM (n = 3). ns: not significant.
(TIF)

S1 Text Supporting materials, methods and references. (DOCX)

S1 Table List and sequence of primers used in this study. (PDF)

S2 Table List of *Ae*. *aegypti* miRNAs that have significant potential to bind to the 5’ or 3’ UTR of the DENV genome. The target sequence in the 5’ or 3’ UTR, and the name of the miRNA are given, followed by the MFE (in kcal/mol) of the best match of the miRNA against the real target sequence. The third column shows the range of MFEs of the best matches of the miRNA against simulated target sequences, followed by the percentage of simulated MFEs, marked by asterisks.
(DOCX)
