# Supplementary figures and images for: Wolbachia-mediated virus blocking in mosquito cells is dependent on XRN1-mediated viral RNA degradation and influenced by viral replication rate
Source: PLoS Pathog. 2018 Mar 1;14(3):e1006879. doi: 10.1371/journal.ppat.1006879 (PMC5833283; doi:10.1371/journal.ppat.1006879)

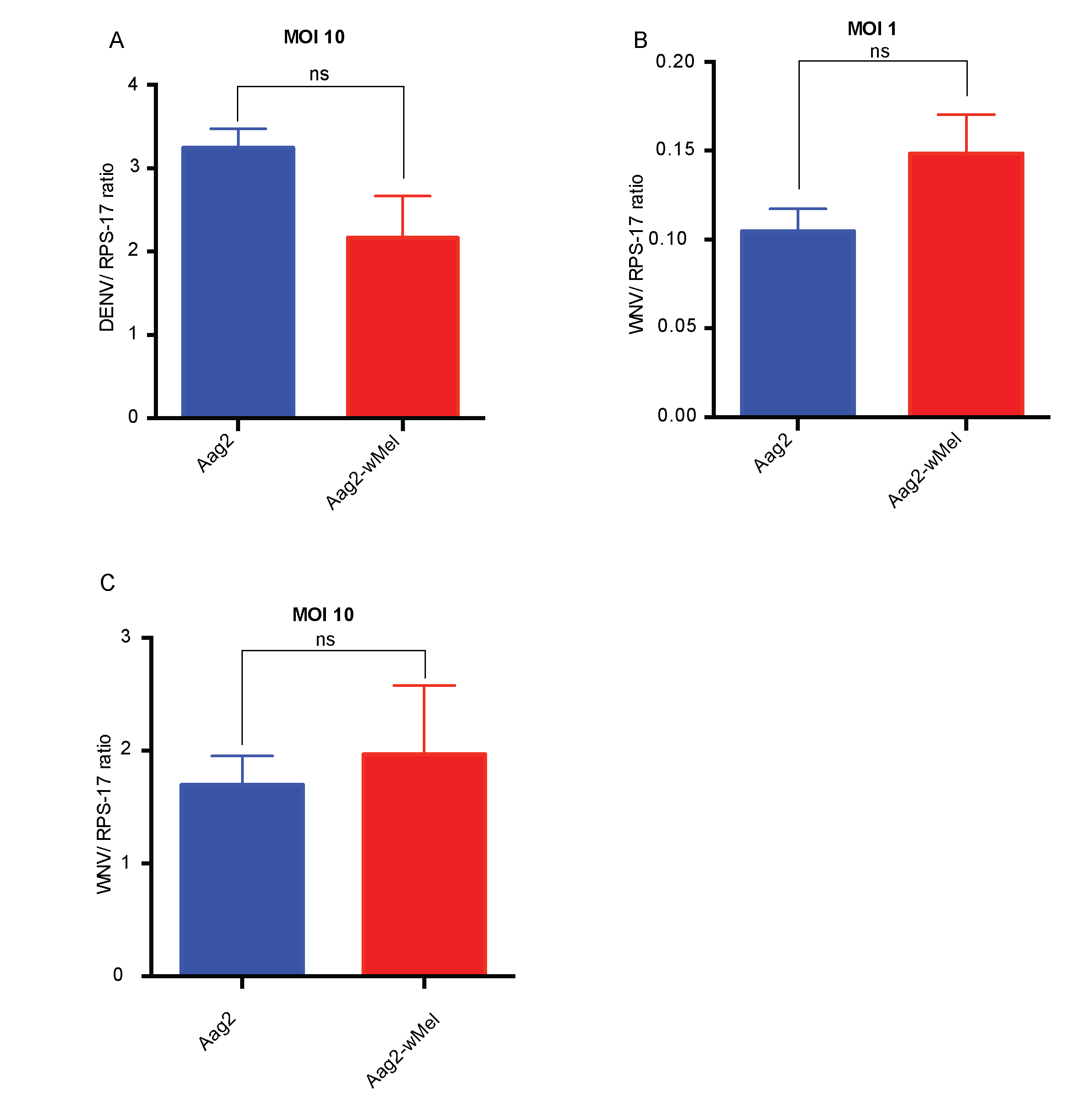

Supplement: S1 Fig — (A) Aag2 and Aag2-wMel cells were infected with DENV at an MOI of 10 at 4°C. RNA levels determined from total cellular RNA through quantitative RT-qPCR using primers DENV-G-F, DENV-G-R and DENV-G- FAM Probe and normalised to RPS-17 RNA levels using primers Rps17_TaqM_FW, Rps17_TaqM_RV, and rps17-LC640 probe. Data are expressed as mean ± SEM (n = 3). ns: not significant. (B &C) Aag2 & Aag2-wMel cells were infected with WNV at an MOI of 1 and 10 at 4°C. RNA levels determined from total cellular RNA through quantitative SYBR Green RT-qPCR using primers WNKUNJ3UTR-F and WNKUNJ3UTR-R and normalised to RPS-17 RNA levels using primers Rps17_TaqM_FW, Rps17_TaqM_RV. Data are expressed as mean ± SEM (n = 3). ns: not significant. (TIF) [file ppat.1006879.s001.tif]

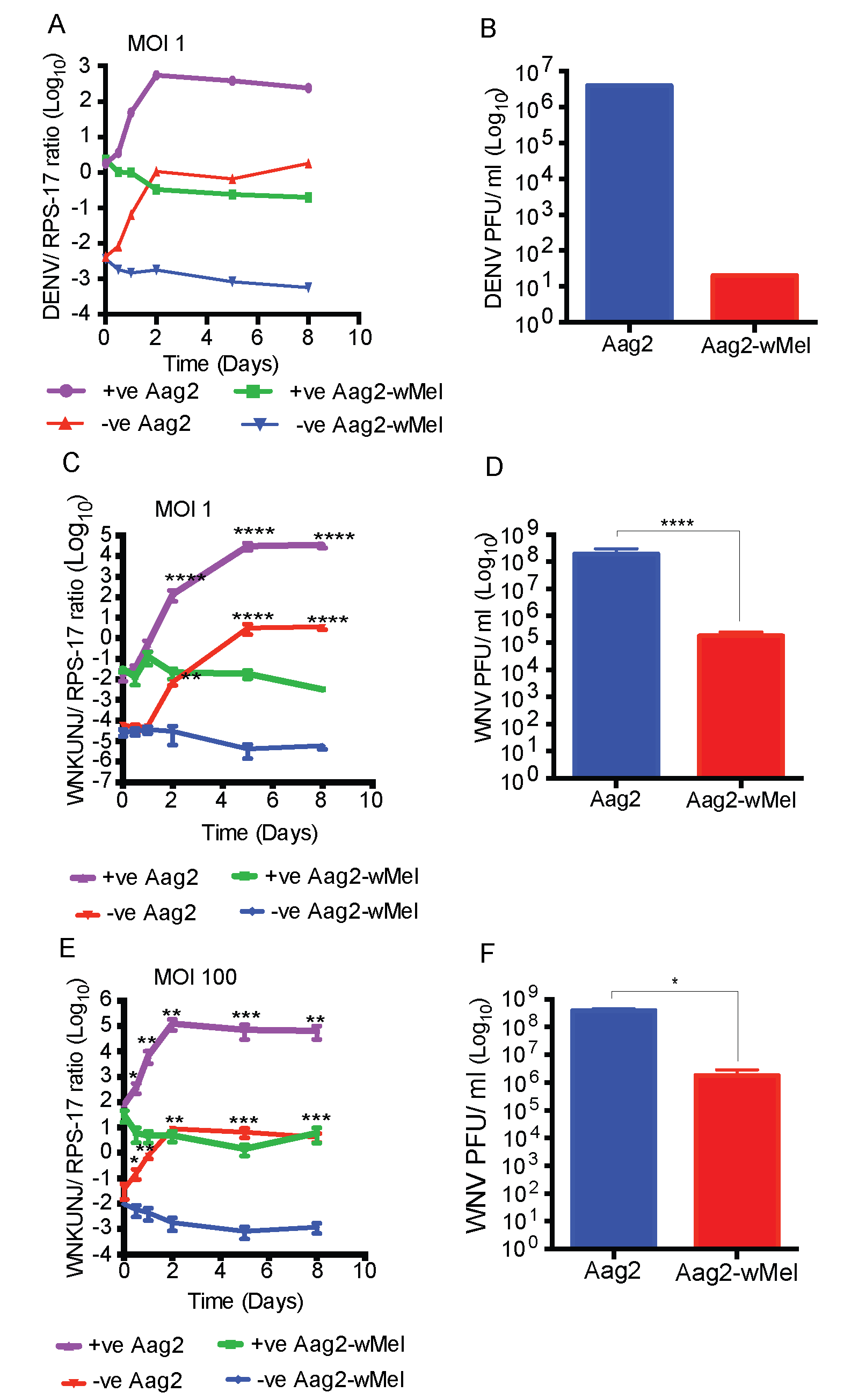

Supplement: S2 Fig — (A) Aag2 & Aag2-wMel cells were infected with DENV (MOI = 1) to determine DENV positive and negative strands, levels at different time points from total cellular RNA through quantitative RT-qPCR and normalised to RPS-17 RNA levels. Data are expressed as mean ± SEM (n = 1). (B) At 5 dpi with DENV, cell culture supernatant was harvested and assayed for viral titre by plaque assay and plotted as plaque forming units/ml. (C & E) WNV positive and negative strands, levels quantified through quantitative RT-qPCR after infecting cells MOI = 1, n = 5 (C) and MOI 100, n = 3 (E) and collected at different time points. (D & F) At 5 dpi with WNV, cell supernatant was harvested and assayed for viral titre by plaque assay and plotted as plaque forming units/ml MOI = 1, n = 5 (D) and MOI 100, n = 3 (F). All Data are expressed as mean ± SEM. ns: not significant, * P ≤ 0.05 **P ≤ 0.01, *** P ≤ 0.001, ****P ≤ 0.0001. (TIF) [file ppat.1006879.s002.tif]

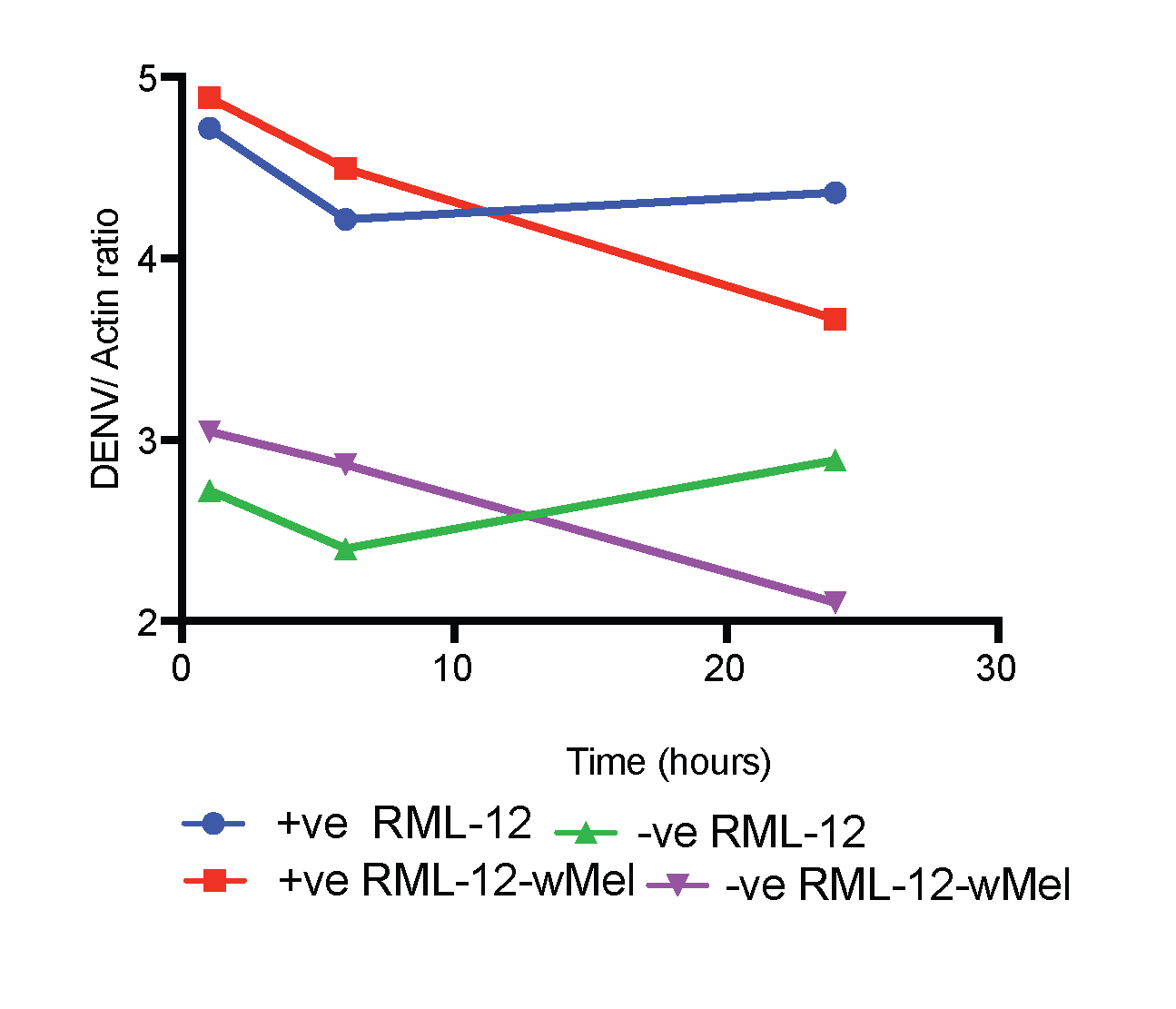

Supplement: S3 Fig — RML-12 and RML-12-wMel cells were infected with DENV (MOI = 1) to determine DENV positive and negative strands, levels at different time points from total cellular RNA through quantitative RT-qPCR and normalised to Actin RNA levels (n = 1). (TIF) [file ppat.1006879.s003.tif]

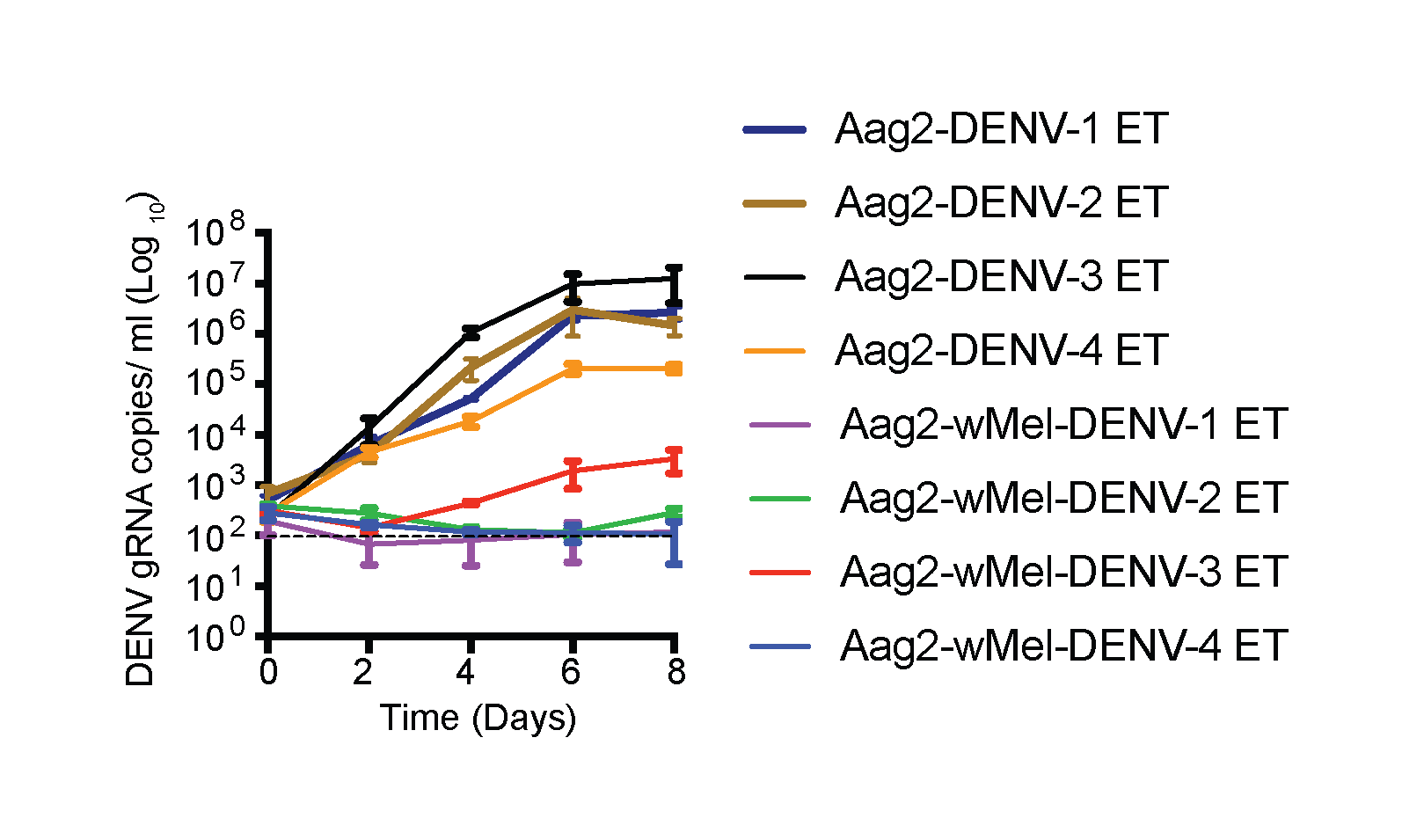

Supplement: S4 Fig — Viral RNA was isolated from cell culture supernatant at different time points and the number of virus copies were calculated using RT-qPCR. Dotted line denotes the minimum detection limit. Data are expressed as mean ± SEM (n = 3). (TIF) [file ppat.1006879.s004.tif]

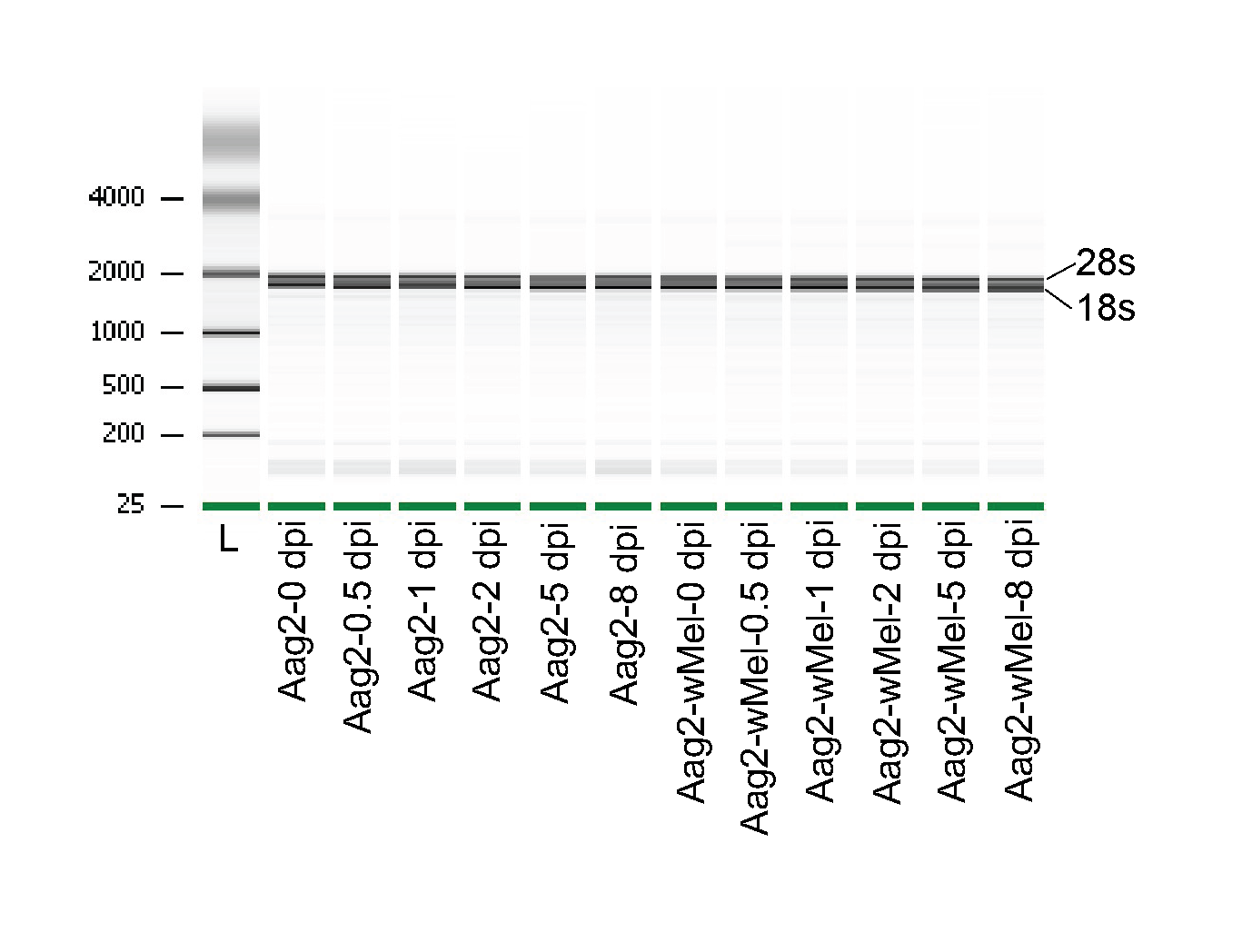

Supplement: S5 Fig — Purified total RNA samples from Aag2 and Aag2 wMel cells were examined using BioAnalyzer 2100 (Aglient Technologies) to check RNA integrity. Major ribosomal RNA bands are indicated. (TIF) [file ppat.1006879.s005.tif]

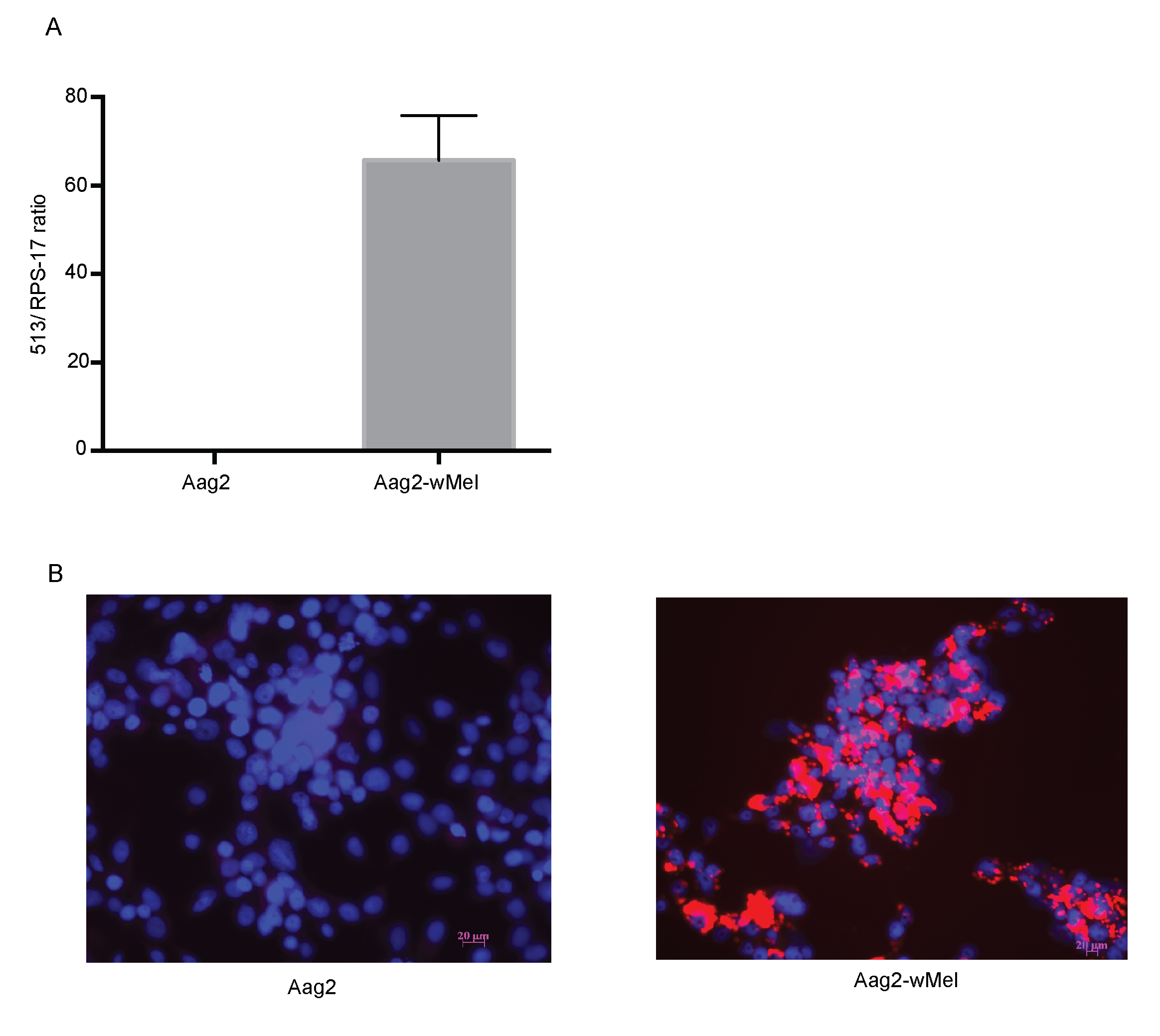

Supplement: S6 Fig — (A)Wolbachia density was calculated through qPCR as a ratio of the WD_513 gene in Wolbachia normalised to host RPS-17 gene. (B) Wolbachia was detected through Fluorescence in situ hybridization (FISH) shown in red and nucleus stained with DAPI (Blue). (TIF) [file ppat.1006879.s006.tif]

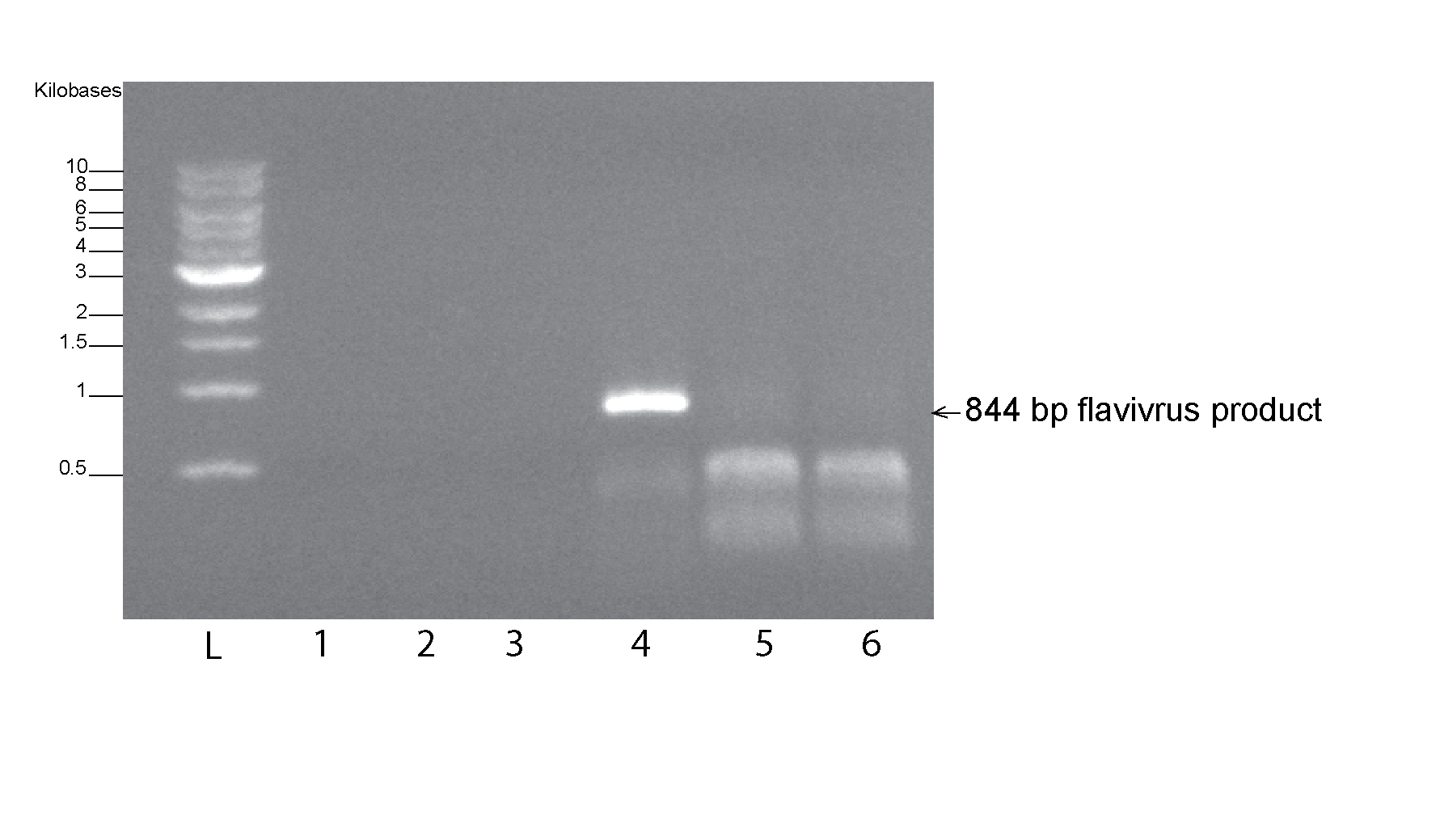

Supplement: S7 Fig — Lane L: 1000 bp DNA ladder; 1: Cell culture supernatant from Aag2; 2: Cell culture supernatant from Aag2-wMel; 3: Water control; 4: Cell culture supernatant from Aag2 cells infected with WNV and harvested 2dpi; 5: Aag2 cells genomic DNA; 6: Aag2-wMel cells genomic DNA. Expected flavirus PCR product is indicated by an arrow. (TIF) [file ppat.1006879.s007.tif]

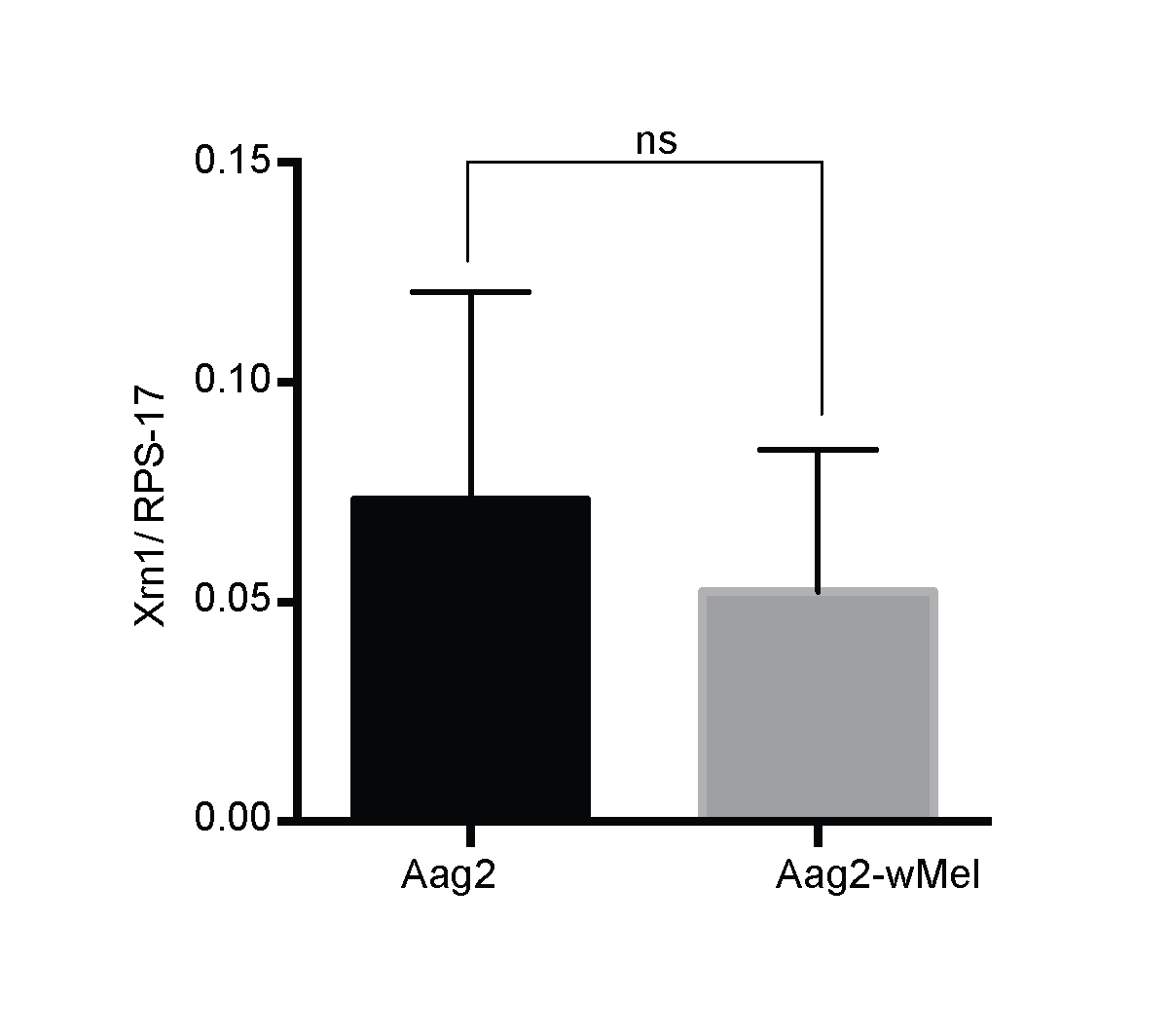

Supplement: S8 Fig — Total cellular RNA five days post DENV infection (MOI = 10) was analysed for expression of XRN1 RNA through quantitative SYBR Green RT-PCR using primers aae-XRN1-Fand aae-XRN1-R normalised to RPS-17 RNA levels. Data are expressed as mean ± SEM (n = 3). ns: not significant. (TIF) [file ppat.1006879.s008.tif]
