# Supplementary material for: Wolbachia-mediated virus blocking in mosquito cells is dependent on XRN1-mediated viral RNA degradation and influenced by viral replication rate
Source: PLoS Pathog. 2018 Mar 1;14(3):e1006879. doi: 10.1371/journal.ppat.1006879 (PMC5833283; doi:10.1371/journal.ppat.1006879)
Supplement: S1 Text — (DOCX) [file ppat.1006879.s009.docx]

**S1 Text**

**Supporting materials and methods**

**Preparation of Aag2-*w*Mel cells**

RML-12 cells of *Aedes albopictus* origin, that were already infected with the *w*Mel strain of *Wolbachia* and maintained in the lab were used for purifying *Wolbachia* and infecting Aag2 cells. To extract *Wolbachia,* RML12-*w*Mel cells were collected in 50 ml falcon tubes and resuspended in SPG buffer containing 218 mM sucrose, 3.8 mM KH_2_PO_4,_ 7.2 mM K_2_HPO_4_, 4.9 mM L- glutamate at pH 7.4. Cells were then sonicated on ice for 2X10 seconds followed by centrifugation at 1000g for 10 minutes at 4 °C. The supernatant was then passed through 5 μm and 2.7 μm filters and centrifuged at 13800 g for 15 minutes at 4 °C to pellet *Wolbachia*.

To infect Aag2 cells with purified *Wolbachia*, Aag2 cells were seeded in 24 well plates overnight. Purified *Wolbachia* was added to the cells and was centrifuged at 1500 g for 1 hour at 26 °C and further incubated at 26 °C overnight. Cells were maintained in complete media and passaged regularly. The density of Wolbachia was calculated by qPCR and FISH.

**Density calculation of *Wolbachia* in cell lines by qPCR.** DNA extracted from Aag2 or Aag2-*w*Mel cells were used to do multiplex qPCR using primers specific to Wolbachia TM513_F 5’-CAAATTGCTCTTGTCCTGTGG-3’, TM513_R GGGTGTTAAGCAGAGTTACGG and TM513 probe Cy5-TGAAATGGAAAAATTGGCGAGGTGTAGG-BHQ3 and Housekeeping gene Rps17_TaqM_FW TCCGTGGTATCTCCATCAAGCT, Rps17_TaqM_RV CACTTCCGGCACGTAGTTGTC, Rps17_TaqM_Probe FAM-CAGGAGGAGGAACGTGAGCGCAG- BHQ1.

qPCR was run using LightCycler® 480 Probes Master (Roche) in a LightCycler® 480 real-time PCR machine with 45 cycles. To find the density 513 gene was normalised with the RPS-17 gene. RML-12 cells were analysed using TM513 primers and normalised with actin primers [1].

**Fluorescence in situ hybridization (FISH)**

Cells were grown overnight on chambered and NUNC slides and processed as described before [2, 3]

***In vitro* transcription of viral RNA**

DENV plasmid containing an infectious clone of DENV-NGC was linearised using XbaI and in vitro transcribed with MEGAscript T7 Transcription Kit (Ambion) along with m7G(5')ppp(5')G RNA Cap Structure Analog (New England Biolabs) according to manufactures instruction. Dnase treated RNA was then transfected into C6/36 cells using Lipofectamine MessengerMAX Transfection Reagent (Invitrogen). Virus produced was isolated from media which was further amplified in C6/36 cells and stored in -80 °C.

**Analysis of cell lines for flavivirus infection**

Aag2 and Aag2–wMel cells were seeded in 12 well plates and either mock infected or infected with Kunjin virus at an MOI of 1. Media and cells were collected 2dpi. The virus was isolated from the media using QIAamp Viral RNA Mini Kit (qiagen). Viral RNA was DNase (Roche) treated to remove any genomic DNA contamination and converted to cDNA using SuperScript III Reverse Transcriptase (Invitrogen) and PCR was performed using primers FU2 (F) and cFD3 (R) as described before [4]. Media from Aag2 cells infected with Kunjin virus was used as a positive control along with DNA extracted from cell lines to look for any non-specific amplification as Aag2 cells genome harbour various flavivirus integrated sequences [5]. PCR product was run in 1% agaorse gel stained with RedSafe (Intron biotechnology).

**Supplementary references**

1. Kwon H, Lu HL, Longnecker MT, Pietrantonio PV. Role in diuresis of a calcitonin receptor (GPRCAL1) expressed in a distal-proximal gradient in renal organs of the mosquito Aedes aegypti (L.). PloS one. 2012;7(11):e50374. doi: 10.1371/journal.pone.0050374. PubMed PMID: 23209727; PubMed Central PMCID: PMCPMC3510207.

2. Frentiu FD, Robinson J, Young PR, McGraw EA, O'Neill SL. Wolbachia-mediated resistance to dengue virus infection and death at the cellular level. PloS one. 2010;5(10):e13398. doi: 10.1371/journal.pone.0013398. PubMed PMID: 20976219; PubMed Central PMCID: PMC2955527.

3. Moreira LA, Iturbe-Ormaetxe I, Jeffery JA, Lu G, Pyke AT, Hedges LM, et al. A Wolbachia symbiont in Aedes aegypti limits infection with dengue, Chikungunya, and Plasmodium. Cell. 2009;139(7):1268-78. doi: 10.1016/j.cell.2009.11.042. PubMed PMID: 20064373.

4. Kuno G, Chang GJ, Tsuchiya KR, Karabatsos N, Cropp CB. Phylogeny of the genus Flavivirus. Journal of virology. 1998;72(1):73-83. PubMed PMID: 9420202; PubMed Central PMCID: PMC109351.

5. Miesen P, Joosten J, van Rij RP. PIWIs Go Viral: Arbovirus-Derived piRNAs in Vector Mosquitoes. PLoS pathogens. 2016;12(12):e1006017. doi: 10.1371/journal.ppat.1006017. PubMed PMID: 28033427; PubMed Central PMCID: PMC5198996.
