# Supplementary material for: Wolbachia-mediated virus blocking in mosquito cells is dependent on XRN1-mediated viral RNA degradation and influenced by viral replication rate
Source: PLoS Pathog. 2018 Mar 1;14(3):e1006879. doi: 10.1371/journal.ppat.1006879 (PMC5833283; doi:10.1371/journal.ppat.1006879)
Supplement: S1 Table — (PDF) [file ppat.1006879.s010.pdf]

No	Name of primer	Sequence	References
1.	DENV-G-F	AAGGACTAGAGGTTAGAGGAGACCC	[1]
2.	DENV-G-R	CGTTCTGTGCCTGGAATGATG	[1]
3.	DENV-G-Probe	FAM-AACAGCATATTGACGCTGGGAGAGACCAGA-BHQ1	[1]
4.	TAGWNKUNJ-E-F	**GGCCGTCATGGTGGCGAATAA**TGGAGGAACAGAGAGACGCTAA (Tag sequence in bold)	This study
5.	TAGWNKUNJ-E-R	**GGCCGTCATGGTGGCGAATAA**TGAGAGCCCAATGCTATCACAG (Tag sequence in bold)	This study
6.	Tag only	GGCCGTCATGGTGGCGAATAA	[2]
7.	WNKUNJ-E-F	TGGAGGAACAGAGAGACGCTAA	This study
8.	WNKUNJ-E-R	TGAGAGCCCAATGCTATCACAG	This study
9.	Rps17_TaqM_FW	TCCGTGGTATCTCCATCAAGCT	[3]
10.	Rps17_TaqM_RV	CACTTCCGGCACGTAGTTGTC	[3]
11.	Rps17-LC640 probe	LC640-CAGGAGGAGGAACGTGAGCGCAG- lowa black	[3]
12.	DENV2_1-28_fw	AGTTGTTAGTCTACGTGGACCGACAAAG	This study
13.	DEN25UTR-R	CCGTTGGTTATTCATCAGAGATCTGCTC	This study
14.	DEN2CP-F	CGAGAGAAACCGCGTGTCAA	This study
15.	DEN2CP-R	AACGAAGGAATGCCACCAGG	This study
16.	DEN2-E-F	CAGGCTATGGCACTGTCACGAT	This study
17.	DEN2-E-R	CCATTTGCAGCAACACCATCTC	This study
18.	DEN2-NS3-F	GCCGGAGTATTGTGGGATGT	This study.
19.	DEN2-NS3-R	GCTCCGATCTGCGAGTATCC	This study
20	WNKUNJ3UTR-F	TCAAGGCCCAATGTCAGACC	This study
21	WNKUNJ3UTR-R	GGGACGTTGATTCGCCTTTG	This study
22.	QG-F	CCATGAAAAGATTCAGAAG	[4]
23.	QGSF-R	GCTGCGATTTGTAAGGG	[4]
24.	QGSF-F	GTGAGCCCCGTCCAAGG	[4]
25.	aae-miR-2a-5p	ACTCTCAAAGTGGTTGTGAAA	This study
26.	aae-miR-8-5p-fw	CATCTTACCGGGCAGCATTAGAAAA	This study
27.	aae-miR-9a	TCTTTGGTTATCTAGCTGTATGA	This study

28.	aae-miR-9b	TCTTTGGTGATTTTAGCTGTATGC	This study
29.	aae-miR-9c-3p	TAAAGCTTTAGTACCAGAGGTC	This study
30.	aae-miR-10	ACCCTGTAGATCCGAATTTGTT	This study
31.	aae-miR-12-5p	CTGAGTATTACATCAGGTACTGGTAA	This study
32.	aae-miR-210-fw	CTTGTGCGTGTGACAACGGAAAA	This study
33.	aae-miR-263b-5p-fw	CTTGGCACTGGGAGAATTCACAGAA	This study
34.	aae-miR-275-3p-fw	TCAGGTACCTGAAGTAGCGCAAAAA	This study
35.	aae-miR-276-5p-fw	CGAGCGAGGTATAGAGTTCCTACAAAAAA	This study
36.	aae-miR-277-5p-fw	CCGTGTCAGAAGTGCATTTACAAAAAAA	This study
37.	aae-miR-281-5p-fw	GAAGAGAGCTATCCGTCGACAAAAAA	This study
38.	aae-miR-285	TAGCACCATTCGAAATCAGTAC	This study
39.	aae-miR-308-3p-fw	GCGGAATCACAGGAGTATACTGAAAAAA	This study
40.	aae-miR-309a-fw	TCACTGGGCAAAGTTTGTCGCA	This study
41.	aae-miR-965	TAAGCGTATAGCTTTTCCCATT	This study
42.	aae-miR-970-fw	GTCATAAGACACACGCGGCTATAAAAA	This study
43.	aae-miR-981	TTCGTTGTCGACGAAACCTGCA	This study
44.	aae-miR-988-5p-fw	CGTGTGCTTTGTGACAATGAGAAAAA	This study
45.	aae-miR-989-fw	GCTGTGATGTGACGTAGTGGTACAAAAAA	This study
46.	aae-miR-998	TAGCACCATGAGATTCAGC	This study
47.	aae-miR-1891-fw	GCTGAGGAGTTAATTTGCGTGTTTAAAAA	This study
48.	Aae-mir-2940-5p-fw	CTGGTTTATCTTATCTGTCGAGGCAAAA	This study
49.	aae-miR-2941-2-fw	CTAGTACGGCTAGAACTCCACGGAAAA	This study
50.	aae-miR-2943-1-fw	GTTAAGTAGGCACTTGCAGGCAAAAA	This study
51.	aae-miR-2945-3p-fw	CGTGACTAGAGGCAGACTCGTTTAAAAAA	This study
52.	aae-bantam-3p	TGAGATCATTTTGAAAGCTGAT	This study
53.	aae-let-7-fw	GCGTGAGGTAGTTGGTTGTATAGTAAAAAA	This study
54.	aae-XRN1-F	ACGGGAACTACTCGGTCGTA	This study
55.	aae-XRN1-R	GTTCTCCCGTGGTTCACGAT	This study
56.	aae-ECR-F	GATCTATCGCCTTCCAGCAG	This study
57.	aae-ECR-R	GCAGGTGAGGGCATTGTAGT	This study

58.	aae-La-F	GATCAGCGAGGACCGTGAAA	This study
59.	aae-La-R	CGCTCATCTGAGTACCCTCC	This study
60.	WNKUNJ3UTR-F	TCAAGGCCCAATGTCAGACC	This study
61.	WNKUNJ3UTR-R	GGGACGTTGATTCGCCTTTG	This study
62.	TM513_F	CAAATTGCTCTTGTCCTGTGG	[5]
63.	TM513_R	GGGTGTTAAGCAGAGTTACGG	[5]
64.	TM513 Probe	Cy5-TGAAATGGAAAAATTGGCGAGGTGTAGG-BHQ3	[5]
65.	AA-actin-F	GACTACCTGATGAAGATCCTGAC	[6]
66.	AA-actin-R	GCACAGCTTCTCCTTAATGTCAC	[6]

References

1. Warrilow D, Northill JA, Pyke A, Smith GA. Single rapid TaqMan fluorogenic probe based PCR assay that detects all four dengue serotypes. Journal of medical virology. 2002;66(4):524-8. PubMed PMID: 11857532.
2. Tay MY, Saw WG, Zhao Y, Chan KW, Singh D, Chong Y, et al. The C-terminal 50 amino acid residues of dengue NS3 protein are important for NS3-NS5 interaction and viral replication. The Journal of biological chemistry. 2015;290(4):2379-94. doi: 10.1074/jbc.M114.607341. PubMed PMID: 25488659; PubMed Central PMCID: PMC4303688.
3. Joubert DA, Walker T, Carrington LB, De Bruyne JT, Kien DH, Hoang Nle T, et al. Establishment of a Wolbachia Superinfection in Aedes aegypti Mosquitoes as a Potential Approach for Future Resistance Management. PLoS pathogens. 2016;12(2):e1005434. doi: 10.1371/journal.ppat.1005434. PubMed PMID: 26891349; PubMed Central PMCID: PMCPMC4758728.
4. Bidet K, Dadlani D, Garcia-Blanco MA. G3BP1, G3BP2 and CAPRIN1 are required for translation of interferon stimulated mRNAs and are targeted by a dengue virus non-coding RNA. PLoS pathogens. 2014;10(7):e1004242. doi: 10.1371/journal.ppat.1004242. PubMed PMID: 24992036; PubMed Central PMCID: PMC4081823.
5. Joubert DA, O'Neill SL. Comparison of Stable and Transient Wolbachia Infection Models in Aedes aegypti to Block Dengue and West Nile Viruses. PLoS neglected tropical diseases. 2017;11(1):e0005275. doi: 10.1371/journal.pntd.0005275. PubMed PMID: 28052065; PubMed Central PMCID: PMCPMC5241016.
6. Kwon H, Lu HL, Longnecker MT, Pietrantonio PV. Role in diuresis of a calcitonin receptor (GPRCAL1) expressed in a distal-proximal gradient in renal organs of the mosquito Aedes aegypti (L.). PloS one. 2012;7(11):e50374. doi: 10.1371/journal.pone.0050374. PubMed PMID: 23209727; PubMed Central PMCID: PMCPMC3510207.
